# Quantifying energetic and fitness consequences of seasonal heterothermy in an Arctic ungulate

**DOI:** 10.1002/ece3.7049

**Published:** 2020-11-22

**Authors:** Jean‐Pierre Desforges, Floris M. van Beest, Gonçalo M. Marques, Stine H. Pedersen, Larissa T. Beumer, Marianna Chimienti, Niels Martin Schmidt

**Affiliations:** ^1^ Bioscience Department Aarhus University Roskilde Denmark; ^2^ Arctic Research Centre Aarhus University Aarhus Denmark; ^3^ Department of Natural Resource Sciences McGill University Ste‐Anne‐de‐Bellevue QC Canada; ^4^ Marine, Environment & Technology Center (MARETEC) Instituto Superior Técnico Universidade de Lisboa Lisboa Portugal; ^5^ Department of Biological Sciences University of Alaska Anchorage Anchorage AK USA; ^6^ Cooperative Institute for Research in the Atmosphere Colorado State University Fort Collins CO USA

**Keywords:** dynamic energy budget, metabolic rate, muskox (*Ovibos moschatus*), reproduction, thermal physiology

## Abstract

Animals have adapted behavioral and physiological strategies to conserve energy during periods of adverse conditions. Heterothermy is one such adaptation used by endotherms. While heterothermy—fluctuations in body temperature and metabolic rate—has been shown in large vertebrates, little is known of the costs and benefits of this strategy, both in terms of energy and in terms of fitness. Hence, our objective was to model the energetics of seasonal heterothermy in the largest Arctic ungulate, the muskox (*Ovibos moschatus*), using an individual‐based energy budget model of metabolic physiology. We found that the empirically based drop in body temperature (winter max ~−0.8°C) overwinter in adult females resulted in substantial fitness benefits in terms of reduced daily energy expenditure and body mass loss. Body mass and energy reserves were 8.98% and 14.46% greater in modeled heterotherms compared to normotherms by end of winter. Based on environmental simulations, we show that seasonal heterothermy can, to some extent, buffer the negative consequences of poor prewinter body condition or reduced winter food accessibility, leading to greater winter survival (+20%–30%) and spring energy reserves (+10%–30%), and thus increased probability of future reproductive success. These results indicate substantial adaptive short‐term benefits of seasonal heterothermy at the individual level, with potential implications for long‐term population dynamics in highly seasonal environments.

## Graphical abstract

Animals use seasonal heterothermy to conserve energy during periods of adverse environmental conditions. Our energy budget model of muskoxen reveals considerable fitness benefits of overwinter heterothermy in the form of conserved body mass, energy reserves, and increased survival, relative to simulated normothermic individuals. Individual energetics and fitness consequences of heterothermy could have implications for population dynamics. Photo credit: Lars Holst Hansen.

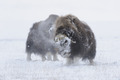

## 1 INTRODUCTION

In the wild, animals often need to contend with variable and harsh environmental conditions. This is particularly true for seasonal and unpredictable environments, where climatic conditions can change drastically over the course of the year. Seasonal changes in external conditions, especially ambient temperature and food availability, have important consequences for endotherms as they use energy to maintain high and stable body temperatures for optimal physiological function (Angilletta et al., 2010). Indeed, temperature regulation can be energetically costly, and energetic stress becomes compounded during periods of cold temperatures and resource scarcity (Geiser, 2004; Ruf & Geiser, 2015). For endothermic species that do not migrate over vast distances, behavioral and physiological adaptations are the only available strategies to minimize exposure to local conditions and to mitigate the impacts of energy limitation (Signer et al., 2011). Species can reduce their energy expenditure by adjusting locomotor activity, body size, body temperature, and metabolic rates (Arnold et al., 2006; Brinkmann et al., 2012; Dehnel, 1949; Riek et al., 2017). Reduced body temperature (*T_b_*) and metabolic rate associated with heterothermy and hypometabolism can range from long‐term hibernation to daily torpor (Geiser, 2004).

Heterothermic mammals abandon strict normothermy and reduce *T_b_* and metabolic rate, which provides the means to reduce energy expenditure during periods of food shortage and thus increases the chance of survival and poststress energy reserves (Humphries et al., 2003; Ruf & Geiser, 2015). Temporal or seasonal heterothermy has been demonstrated for virtually all animal phyla and broadly across latitudinal gradients, highlighting the evolutionary and ecological significance of metabolic flexibility in response to changing environments (e.g., Angilletta et al., 2010; Boyles et al., 2013; Geiser, 2004; Guppy & Withers, 1999; McKechnie & Mzilikazi, 2011). Because of opposing energetic demands, heterothermy and reproduction in some mammals may be mutually exclusive processes, leading to reproductive periods in spring and summer followed by periods of heterothermy in autumn and winter, with no temporal overlap in the two states (McAllan & Geiser, 2014). For many northern species, for example, mountain goats, arctic voles and lemmings, wolverines, reindeer, and muskox (Arnold et al., 2018; Bronson, 2009; Schmidt et al., 2020; Thiel et al., 2019), costly reproductive processes of gestation and lactation overlap with the food‐restricted winter season, when energetic stress is already at its greatest due to high thermoregulatory costs and low food availability. These winter conditions have been shown to be a major determinant of body condition, reproduction, and population dynamics in mammals, such as northern ungulates, living in harsh environments (Gaillard et al., 2000; Helle & Kojola, 2008; Schmidt et al., 2015), highlighting the potential impact of seasonal environments on animal fitness.

Arctic and alpine ecosystems undergo some of the most extreme seasonal shifts in environmental conditions, with dramatic fluctuations in food availability and quality, temperature, light, and precipitation. Terrestrial arctic and alpine animal species depend on short snow‐free periods to maximize intake of high quality and abundant forage to replenish energy reserves lost during the preceding resource‐scarce winter months and gain sufficient stores for the following winter. Although empirical evidence of lowered *T_b_* in free‐ranging Arctic and alpine mammals as an energy‐saving strategy to adverse winter conditions is growing (Arnold et al., 2018; Signer et al., 2011; Thiel et al., 2019), quantifications of related energy savings and possible fitness consequences are lacking. Further, individual phenotypic variation in the degree of heterothermy has been shown to be important for intrapopulation fitness (e.g., Dammhahn et al., 2017). As such, investigating the energetic and fitness implications of different overwintering thermoregulating strategies for species living in harsh seasonal environments, such as the Arctic, is important to understand the ecological implications of heterothermy.

**FIGURE 1:**
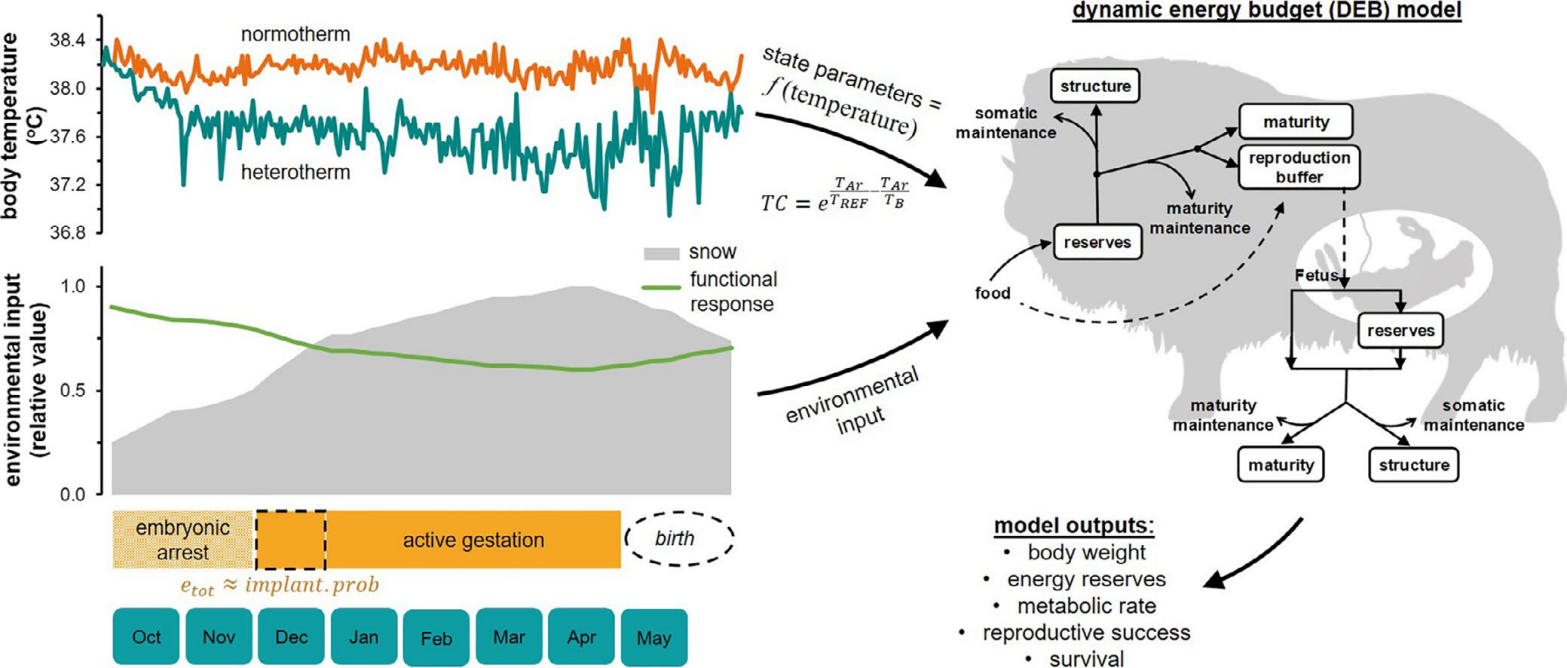
Schematic representation of the dynamic energy budget linked individual‐based model (DEB‐IBM) for muskoxen. Body temperature (TB) influences the energy budget through a temperature correction factor (TC) on metabolic rates. Outlined (dashed) periods in the model schedule represent stochastic events determined probabilistically according to noted parameters. The schematic DEB model of energy intake and allocation to metabolic processes was taken from Desforges et al. (2019)

As a study organism, we use the muskox (*Ovibos moschatus*), a capital breeding species living in the high Arctic that uses energy reserves accrued in summer to increase survival and cover costly reproductive needs associated with gestation and lactation in winter (Figure 1). We aim to quantify the consequences of seasonal heterothermy on animal fitness using a systematic theoretical approach by comparing daily and cumulative individual responses in seasonally normothermic versus heterothermic animals using an individual‐based dynamic energy budget model (DEB‐IBM). Specifically, we ask the question: What are the potential energetic benefits of winter heterothermy? We also test the influence of physiological and environmental factors on the consequences of different overwinter metabolic strategies using a suite of environmental simulations.

## 2 MATERIAL AND METHODS

### 2.1 Study species

Muskoxen are the largest herbivore in the Arctic and as such play a crucial role in ecosystem form and function (Kutz et al., 2017). The study population is located in the Zackenberg valley, Northeast Greenland (74°28′N; 21°33′W) and is a focal species in the long‐term Zackenberg Basic monitoring program in high arctic Greenland (Schmidt et al., 2015). The high Arctic climate is characterized by a mean annual air temperature of –9°C and mean annual precipitation of 260 mm, falling primarily as snow (Hansen et al., 2008). The Arctic summer provides approximately 3 months of high quality forage, while the snow‐dominated winter months allow minimal access to low quality forage for the rest of the year. Snow conditions are thus a major determinant of muskox movement ecology and population dynamics (Schmidt et al., 2015, 2016). As a capital breeder, muskoxen rely on energy reserves accumulated throughout the short summer season to fuel costly reproductive processes (gestation and lactation; Desforges et al., 2019) and somatic maintenance needs the following winter and spring (Figure 1a).

A recent study on muskoxen in northeast Greenland found that nonpregnant adult females exhibited overwinter heterothermy, measured as declining $T_{B}$ throughout winter, whereas pregnant females maintained relatively stable $T_{B}$ over the same time period and region (Schmidt et al., 2020). Together with previous reports of lower organ weights, energy expenditure, and reduced maintenance needs during winter (Adamczewski et al., 1997; Lawler & White, 1997), it is evident that muskoxen adopt physiological strategies to conserve energy during harsh winter months. We used the data on $T_{B}$ from Schmidt et al. (2020) to model the potential energetics and fitness consequences of seasonal heterothermy in muskoxen. For simplicity, we grouped animals into two categories that represent diverging seasonal patterns of $T_{B}$, namely normotherms (relatively stable $T_{B}$; winter mean 38.2°C) and heterotherms (reduced overwinter $T_{B}$; overwinter mean 37.4°C) (Figure 1a). These correspond to ‘survived, birth (wild)’ (normotherms, *n* = 3) and ‘survived, no birth (wild)’ (heterotherms, *n* = 2) animals in Schmidt et al. (2020). We acknowledge that both groups of animals are characterized by temporal variability in $T_{B}$ and thus could be considered along a heterothermy continuum (Boyles et al., 2013), but we use these simplified groupings herein to represent broad seasonal thermoregulatory patterns.

### 2.2 Model overview

We used a DEB‐IBM to characterize the metabolic physiology of adult female muskoxen in northeast Greenland. All aspects of animal energetics were modeled using dynamic energy budget (DEB) theory for metabolic organization (Kooijman, 2010). Briefly, assimilated energy from the environment is accumulated in reserves, then mobilized following the kappa rule to fuel somatic maintenance and growth (kappa branch) and maturity maintenance, maturation, and reproduction (1 – kappa branch). Because detailed winter food intake is unknown, we modeled food acquisition in muskoxen as an inverse function of snow depth (see details below). A schematic representation of the DEB model is shown in Figure 1b, and the model is fully described in Appendix S1 and general aspects previously reported in Desforges et al. (2019). The model was implemented in NetLogo (version 6.0.2, 4 August 2017) and results analyzed and illustrated in R (R Core Team, 2020).

Our experimental design was focused on the systematic paired comparison of normothermic and heterothermic overwinter $T_{B}$ profiles as they relate to individual fitness. The model targeted the period of the year most influential to muskox population dynamics, namely the end of summer to early spring (i.e., overwinter period) when $T_{B}$ has been observed to fluctuate most between normothermic and heterothermic animals (Schmidt et al., 2020). The model used a daily time step over the simulation period of October to May, which covered the period of embryonic arrest, active gestation, and birth as well as the period with highest snow accumulations and least amount of forage (Figure 1a). Pregnant females had lower average body mass than nonpregnant (Schmidt et al., 2020), but similar body condition score based on condition index (N.M. Schmidt, Pers. Comm.). Body condition is likely to influence heterothermy in mammals (Humphries et al., 2003), but since the reason for the difference in observed body mass between groups was unknown but not likely due to body condition, we adopted the simplest model approach to reduce confounding factors: we assumed that normotherms and heterotherms differ only in their $T_{B}$; thus, initial body mass and condition were the same between groups.

We assumed that the energy savings of seasonal heterothermy arose from passive thermal effects of lowered $T_{B}$, via so‐called $Q_{10}$ or Arrhenius effects whereby the rates of enzymes are slowed at lower temperatures (Guppy & Withers, 1999). This is modeled via a temperature correction factor (TC) applied to state parameters that regulate the metabolic rate of individuals in DEB theory (Kooijman, 2010). These included the following rate parameters: volume‐specific somatic maintenance rate ($\left[{\dot{p}}_{M}\right]$), maturity maintenance rate constant (${\dot{k}}_{J}$), and energy conductance ($\dot{v}$). TC was modeled as a function of $T_{B}$, the Arrhenius temperature of the species ($T_{\mathrm{Ar}}$), and a reference temperature ($T_{\mathrm{REF}}$) following:

$$TC=\exp^{\frac{T_{\mathrm{Ar}}}{T_{\mathrm{REF}}}‐\frac{T_{\mathrm{Ar}}}{T_{B}}}$$

$T_{\mathrm{REF}}$ is a reference temperature, commonly set to room temperature (293.15 K). The $T_{\mathrm{Ar}}$ of muskoxen in unknown; thus, we use the standard value in DEB theory that was used in the original calibration of the model (Desforges et al., 2019). A sensitivity analysis showed that changes in this value resulted in absolute changes in values (e.g., body mass, reserves), but did not affect the differences between metabolic groups (i.e., normotherm vs. heterotherm) (Figure 6 in Appendix S1). We ignored the influence of ambient temperature on metabolism as muskox are well adapted to freezing temperatures and most likely to be temperature stressed in the summer rather than winter (Munn et al., 2009).

Since it is unclear whether the physiological processes regulating ingestion and assimilation rates are temperature‐dependent (see Discussion for details) and would thus fluctuate with $T_{B}$, or remain constant despite changes in $T_{B}$, we modeled both scenarios. In the model, the maximum specific assimilation rate ($\left\{{\dot{p}}_{\mathrm{Am}}\right\}$) determines the assimilation (i.e., ingestion) rate of the individual and can be temperature corrected. The two tested scenarios were as follows: 1) $\left\{{\dot{p}}_{\mathrm{Am}}\right\}$ is independent of heterothermy (i.e., constant $\left\{{\dot{p}}_{\mathrm{Am}}\right\}$); and 2) $\left\{{\dot{p}}_{\mathrm{Am}}\right\}$ is body temperature‐dependent and thus feeding rates follow reductions in metabolic depression (i.e., temperature corrected $\left\{{\dot{p}}_{\mathrm{Am}}\right\}$).

### 2.3 Model simulations

**FIGURE 2:**
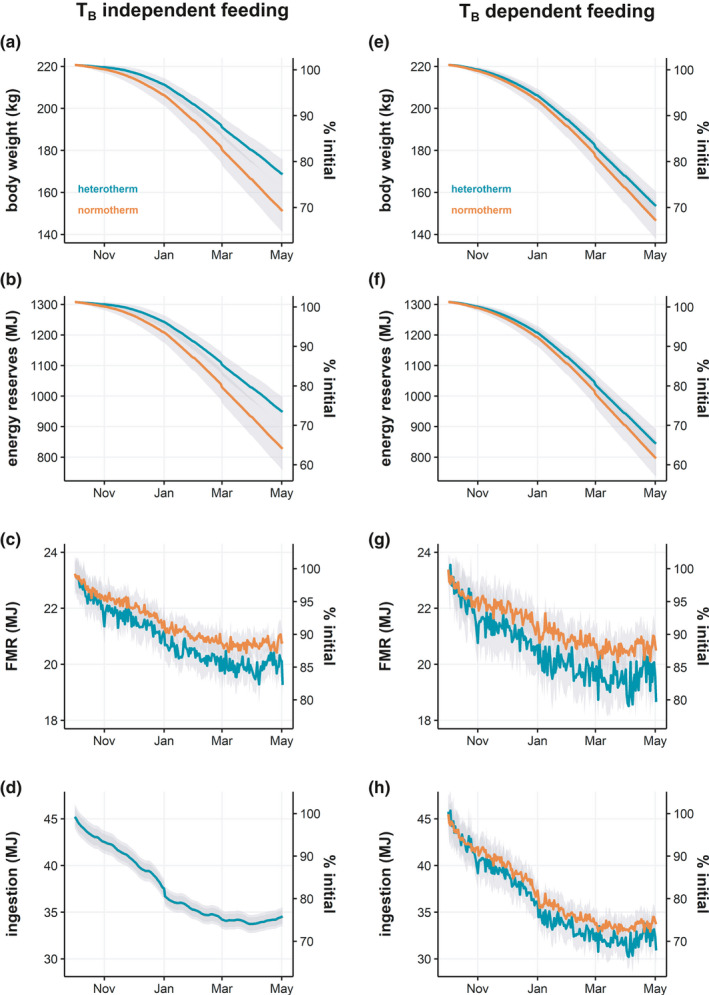
Daily overwinter energetic and fitness profiles of normotherm and heterotherm nonpregnant adult females. Effects of overwinter strategies are shown for (a, e) body weight, (b, f) total energy reserves (sum of reserves and reproduction buffer), (c, g) field metabolic rate (FMR), and (d, f) ingestion rate. Secondary *y*‐axes display the percentage change from starting values. Ingestion is assumed to be body temperature independent in (a‐d) and body temperature dependent in (e‐h). Lines represent simulation means ± standard deviation

We used the mean daily body temperature of normotherms and heterotherms as reported in Schmidt et al. (2020) and implement individual variability around mean group $T_{B}$ in the model using a stochastic multiplier. The stochastic (scatter‐) multiplier is a log‐normally distributed random number with a mean of zero and a defined coefficient of variation (i.e., $scatter‐multiplier=e^{random‐normal0cv}$). Seasonally heterothermic muskoxen were observed to have a greater range of daily $T_{B}$ than normotherms; thus, the coefficients of variation were set at 0.15 and 0.10, respectively, to simulate the levels of variability observed in the original dataset ($T_{B}\times scatter‐multiplier)$. The resulting modeled $T_{B}$ patterns are illustrated in Figure 1a and in with detailed variability in the Appendix S1 (Figure 2).

Because food intake measurements in free‐ranging muskoxen are unknown, we modeled food acquisition (via the functional response, *f*) as a function of winter precipitation. Snow cover and depth have been shown to be a critical determinant of muskoxen and other northern ungulate population dynamics through its impact on food availability and overall quality (e.g., Coulson, 2001; Coulson et al., 2000; Forchhammer et al., 2001; Schmidt et al., 2015). As input of snow conditions, we used modeled local mean daily snow depth (cm) as a proxy for food availability and acquisition throughout winter. These data were produced over the muskox monitoring area in Zackenberg, NE Greenland (Schmidt et al., 2015), by Pedersen et al. (2018) using MicroMet and SnowModel (Liston & Elder, 2006a, 2006b). To capture the typical snow conditions experienced by muskoxen in this area, we take the average daily values over the past 18 years (2000–2018) and use the spatial mean to get one value per day for all animals. We did not apply a snow depth threshold at which animals cannot access food (i.e., *f* = 0), thus assume animals move to forage throughout winter in this spatially implicit model. Further details on input data and how they were utilized are provided in Appendix S1.

Because we wanted to quantify possible energy savings of heterothermy and associated fitness consequences, model outputs of interest included individual body mass, energy reserves, daily energy expenditure (DEE), reproductive success (i.e., birth rate), and adult survival (i.e., % adults surviving winter). Energy reserves in our DEB model included both the reserves and reproduction buffer and the summed value is reported herein. DEE is approximated by an estimate of field metabolic rate (FMR), which is the sum of dissipated energy fluxes and overhead costs associated with somatic and maturity maintenance (${\dot{p}}_{S}+{\dot{p}}_{J}$), assimilation (${\dot{p}}_{A}$), and reproduction (${\dot{p}}_{R}+{\dot{p}}_{D}^{f}$) according to the following equation:

$$FMR={\dot{p}}_{S}+{\dot{p}}_{J}+{\dot{p}}_{A}\left(1‐\kappa_{X}\right)+{\dot{p}}_{R}\left(1‐\kappa_{R}\right)+{\dot{p}}_{D}^{f}$$

with ${\dot{p}}_{D}^{f}$ as the sum of dissipating energy fluxes of the developing embryo. The probability of mortality in adults and embryos in the model is stochastic and determined using logistic function of body condition (scaled total energy reserve density, $e_{\mathrm{tot}}$; see Appendix S1 for details).

While seasonal heterothermy was only observed in the small subset of nonpregnant animals in Schmidt et al. (2020), the presumed benefits of heterothermy extend to improved reproduction via higher energy reserves for reproduction (lactation) the following year. As per our systematic approach, we included pregnancy status as an additional hypothetical factor in the model design. As with nonpregnant females, we modeled seasonal normotherm and heterotherm temperature profiles as two groups of pregnant animals, all else being equal. We included two possible influences of heterothermy on reproduction: (a) embryo homeostasis is preserved and independent of maternal $T_{B}$, and (b) embryo development is a function of maternal $T_{B}$ and thus metabolic rates in the embryo energy budget are temperature corrected. While pregnant muskoxen were not found to adopt heterothermy (Schmidt et al., 2020), the above simulations were included because gestational heterothermy has been reported for other mammals (see review by McAllan & Geiser, 2014) and because it offers the most relevant contrasting scenario (i.e., reduced confounders) to the observed case of gestational normothermy in terms of energetics and fitness.

### 2.4 Environmental simulations

To assess the dynamic costs and benefits of seasonal heterothermy versus normothermy across meaningful biological and ecological conditions, we tested model predictions of adult female fitness in a suite of simulations that captured various aspects of physiological and environmental variability. In the first set of simulations (Scenario 1), we used the same environmental input as the baseline model but assessed the influence of incremental changes in the degree of heterothermy (i.e., change in group means, plus variability). This was modeled as a factor change in the daily values of normotherm $T_{B}$, such that the $T_{B}$ profile of heterotherms followed that of normotherms but with an incremental 0.1°C difference with each time step. The second set of simulations (Scenario 2) was designed to test the effect of initial body condition (i.e., prewinter) using the baseline normotherm and heterotherm $T_{B}$ profiles. Here, we initialized individuals at five percent increments of body reserves, going down from 80% (baseline) to 50% of maximum reserves. This was meant to capture the fact that wild animals are likely to enter winter at different body condition because of reproduction status, summer resource competition, previous harsh winter, etc. The final simulations (Scenario 3) included the effect of changing winter precipitation levels to capture the major winter stress factor for muskoxen, namely food limitation due to snow. This was simulated by incremental changes to the baseline functional response curve (based on baseline daily snow depth), thus increasing or decreasing the mean daily food accessibility by 10%, 20%, and 30%.

### 2.5 Model analysis

All model simulations were run using an arbitrary starting population size of 25 individuals and each simulation was repeated 25 times to capture model stochasticity. All values reported represent the mean of the pooled data (*n* = 25 × 25) ± standard deviation.

## 3 RESULTS

### 3.1 Normothermy versus heterothermy in Arctic muskox

Body mass fell throughout autumn and winter as food accessibility decreased with increasing snow depth. Assuming that feeding rates were not a function of $T_{B}$, seasonal heterothermy led to early and increasing benefits for mitigating body mass loss over the winter. By January, the mean difference in body mass between normotherms and heterotherms was 4.99 ± 1.15 kg, and by May, it increased to 17.34 ± 2.47 kg (Figure 2a). Energy reserves over the model period decreased by 477.15 ± 13.97 MJ and 357.52 ± 9.83 MJ for normotherms and heterotherms, respectively, representing a change from initial summer energy reserves of approximately 36.49% and 27.35%, respectively (Figure 2b). The difference in FMR between groups was negligible in October but grew consistently as the difference in $T_{B}$ increased over the winter, resulting in a 0.65 ± 0.16 MJ/d difference between groups at the end of winter (Figure 2c). Because ingestion was independent of $T_{B}$ in these simulations, the modeled ingestion rates were identical in both groups and varied stochastically (Figure 2d).

When running similar simulations assuming temperature‐dependent effects on state parameters affecting ingestion rates (i.e., $T_{B}$ dependent feeding), the importance of food intake in driving the observed benefits of seasonal heterothermy as previously described becomes evident (Figure 2e‐h). Here, a difference in daily ingestion rate of 1.63 ± 0.41 MJ (Figure 2h) by the end of winter between heterotherms and normotherms due to differences in $T_{B}$ resulted in a 1.12 ± 0.22 MJ/d difference in FMR (Figure 2g) and relatively minor differences in final body mass (6.87 ± 2.22 kg) (Figure 2e) and energy reserves (47.46 ± 1.53 MJ) (Figure 2f).

### 3.2 Normothermy versus heterothermy in pregnant muskox

**FIGURE 3:**
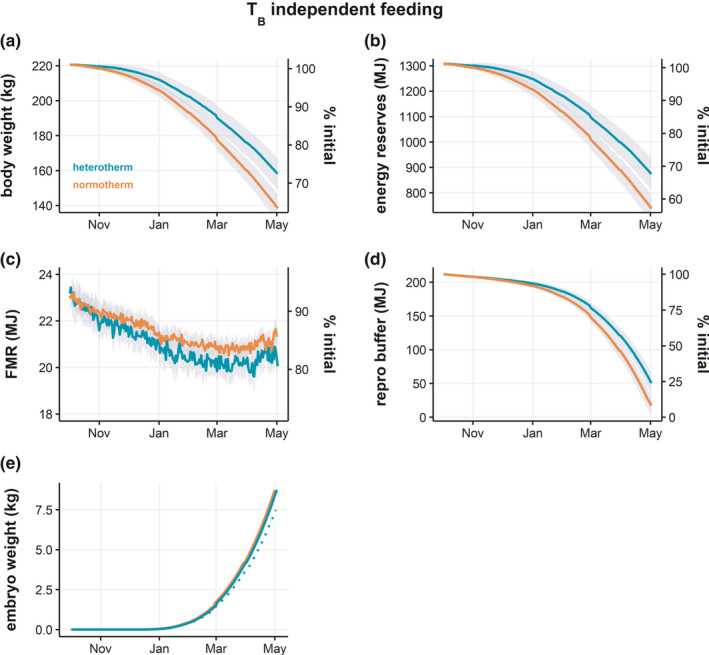
Daily overwinter energetic and fitness profiles of normotherm and heterotherm pregnant adult females assuming ingestion is independent of body temperature. Effects of overwinter strategies are shown for (a) body weight, (b) total energy reserves (sum of reserves and reproduction buffer), (c) field metabolic rate (FMR), (d) reproduction buffer, and (e) embryo growth. Dotted line for embryo growth indicates heterothermic females in which fetal development is a function of maternal body temperature. Secondary *y*‐axes display the percentage change from starting values. Lines represent simulation means ± standard deviation

To better understand the implications of heterothermy on the overwinter fitness of female muskoxen, we modeled the hypothetical case of heterothermy in pregnant animals. Not surprisingly, the difference between normotherms and heterotherms was similar to that in nonpregnant animals, with slightly greater losses in body mass and energy reserves due to the additional costs of gestation (Figure 3a,b). The potential benefit of heterothermy was evident in the energy reserves dedicated for reproduction (i.e., reproduction buffer), where heterotherms had over double the reserves of normotherms (53.19 ± 15.74 MJ vs. 19.62 ± 13.27 MJ) by the end of winter (Figure 3d). When embryo development was modeled as a function of maternal body temperature, calves born to normotherms were 1.22 kg heavier than those born to heterotherms, representing a difference of almost 15% (Figure 3e).

### 3.3 Environmental simulations

**FIGURE 4:**
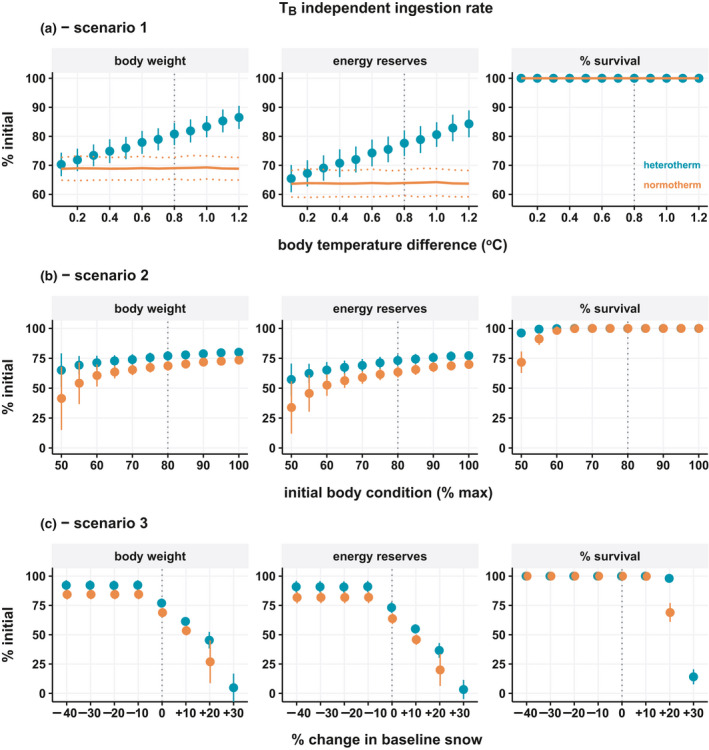
Seasonal energetic and fitness consequence of normothermy and heterothermy in nonpregnant adult females assuming ingestion is independent of body temperature. Body weight, total energy reserves, and adult survival at the end of winter as a percentage of initial values are shown for three model scenarios: (a) Scenario 1—increasing difference in body temperature between groups, (b) Scenario 2—decreasing starting body condition, and (c) Scenario 3—change in daily food accessibility (snow depth) from baseline. Vertical dashed lines indicate baseline model values. Data represent simulation means ± standard deviation

**FIGURE 5:**
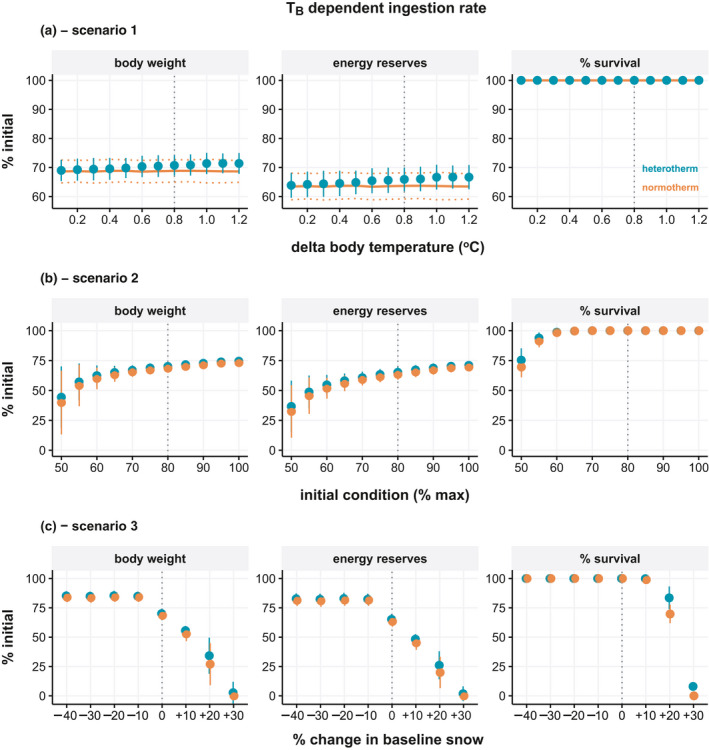
Seasonal energetic and fitness consequence of normothermy and heterothermy in nonpregnant adult females assuming ingestion are dependent of body temperature. Body weight, total energy reserves, and adult survival at the end of winter as a percentage of initial values are shown for three model scenarios: (a) Scenario 1—increasing difference in body temperature between groups, (b) Scenario 2—decreasing starting body condition, and (c) Scenario 3—change in daily food accessibility (snow depth) from baseline. Vertical dashed lines indicate baseline model values. Data represent simulation means ± standard deviation

The first set of environmental simulations (Scenario 1) tested the degree of heterothermy via incremental changes in the daily difference in $T_{B}$ between normotherms and heterotherms. There was a clear and linear effect of $T_{B}$ on body mass and energy reserves, though no effect on survival using baseline seasonal food availability (Figure 4a). With temperature‐dependent feeding, the fitness difference between overwinter strategies becomes minimal and does not increase substantially as the degree of heterothermy increased (Figure 5a).

The second set of simulations (Scenario 2) tested the effect of initial body condition (end of summer) on overwinter fitness. The body condition at the beginning of model simulations had a substantial effect on all fitness endpoints for both groups by the end of winter (Figure 4b). The difference between normotherms and heterotherms increased as initial body condition decreased, and this effect was evident for body mass, energy reserves, and overwinter adult survival. At the lowest starting body condition, overwinter survival was 96.17 ± 3.56% for heterotherms and 71.68 ± 8.98% for normotherms. The benefits of heterothermy for overwinter body mass and energy reserves were again diminished when feeding rates followed metabolic depression as a function of $T_{B}$, with marginal differences apparent only at the lowest initial body condition (Figure 5b).

The third set of simulations (Scenario 3) tested the impact of varying levels of daily overwinter food accessibility by increasing or decreasing snow depth. The baseline snow depth reached a winter maximum of 88 cm, which corresponded to a scaled function response minimum of 0.70. Accordingly, the modeled simulations modified the baseline daily functional response values by factor multiplication, such that the +10%, +20%, +30% scenarios had minimum winter functional responses of 0.63, 0.56, and 0.49 or equivalent to approx. winter maximums of 108 cm, 130 cm, and 150 cm snow, respectively. Survival was already at 100% in the baseline model such that increased food availability as a result of reduced snow, had no effect. However, a daily increase of 10% forage accessibility increased body mass and energy reserves, though the effect was the same for both thermal strategies (Figure 4c). Decreasing forage accessibility, in contrast, negatively affected all fitness endpoints. Heterotherms lost less mass and reserves compared to normotherms, though the differences between groups remained the same across snow simulations. Increasing snow did, however, have a greater impact on survival in normotherms than heterotherms, with 20% less forage accessibility reducing survival to 68.96 ± 8.03% and 98.08 ± 3.02%, respectively. In the most severe scenario, 100% of normotherms and 85.89 ± 6.41% of heterotherms died.

**FIGURE 6:**
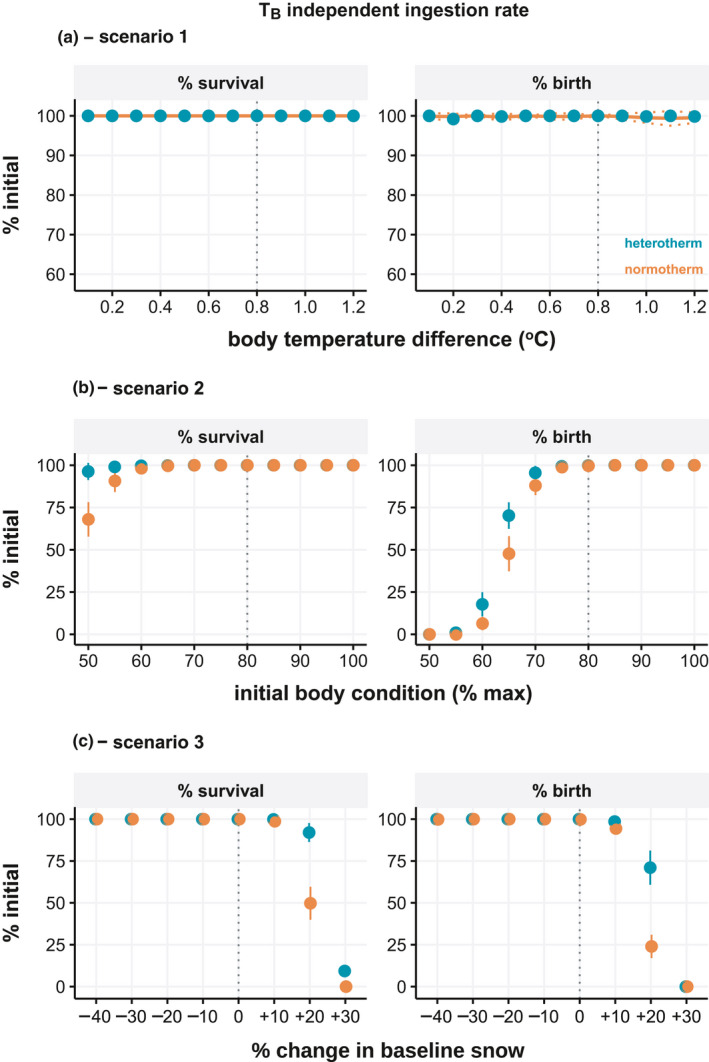
Seasonal energetic and fitness consequence of normothermy and heterothermy in pregnant adult females assuming ingestion are independent of body temperature. Body weight, total energy reserves, and adult survival at the end of winter as a percentage of initial values are shown for three model scenarios: (a) Scenario 1—increasing difference in body temperature between groups, (b) Scenario 2—decreasing starting body condition, and (c) Scenario 3—change in daily food accessibility (snow depth) from baseline. Vertical dashed lines indicate baseline model values. Data represent simulation means ± standard deviation

We repeated the environmental simulations assuming pregnancy in all animals to determine hypothetical implications for reproductive success. Different $T_{B}$ had no effect on survival or reproductive output in pregnant females (Figure 6a). Initial body condition reduced survival at the two lowest conditions tested similar to nonpregnant females, while the effect on birth rate was already evident at 10% lower body condition from baseline and birth rate rapidly dropped at lower starting body conditions; the additional costs of maintaining $T_{B}$ led to lower birth rates compared to heterothermic animals (Figure 6b). Increasing winter precipitation also caused marked reductions in adult survival and birth outcomes (Figure 6c). The difference between groups was most apparent at moderate snow‐reduced forage accessibility (+20%) where 24.00 ± 6.98% and 71.04 ± 10.21% of normotherms and heterotherms gave birth at the end of winter, respectively.

## 4 DISCUSSION

Heterothermy is well documented in small and large mammals (Heldmaier et al., 2004; Ruf & Geiser, 2015) as a means of coping with seasonal and unpredictable variations in ecological conditions (Humphries et al., 2003; Nowack et al., 2017). The primary mechanism at play is reduced energy expenditure with lower $T_{B}$ and metabolic rate, which limits energy reserve depletion to favor both survival and postheterothermy reproduction. For mammals living in harsh seasonal environments like those of the Arctic, heterothermy offers potential life saving energy conservation during resource‐scarce winter months. While some empirical data in free‐ranging and captive large herbivores exist (Arnold et al., 2004, 2006, 2018; Brinkmann et al., 2012; Riek et al., 2017, 2019; Schmidt et al., 2020; Signer et al., 2011; Turbill et al., 2011), these often lack paired metabolic and body mass or energy reserve measurements and are necessarily limited by the thermal regulatory strategy adopted by the study animals. Further, the fitness consequences of heterothermy are difficult to quantify directly. Herein lies the strength of individual‐based modeling approaches as they can provide detailed estimates on energy savings and possible fitness benefits of contrasting behavioral or physiological strategies using available empirical data and first principles (Stillman et al., 2015). Here, we modeled the detailed energetics and associated survival and reproductive performance in a paired group design allowing for a direct quantification of potential benefits of heterothermy as compared to similar animals adopting normothermy, thus eliminating all confounding factors.

### 4.1 Seasonal energetics

We found a marked seasonal impact of heterothermy on the energetics of adult female muskoxen, particularly on overwinter energy reserve depletion and body mass compared to strict normothermy. All individuals were in negative energy balance starting in October as forage quality and quantity decrease as climate shifted to winter temperatures and precipitation. Total mass loss relative to summer highs matched the 40–80 kg noted in free‐ranging Arctic muskoxen (Adamczewski et al., 1992, 1997), and we show energy savings in seasonal heterothermy can result in almost 20 kg of additional body mass by the end of winter relative to strict seasonal normothermy. Since food intake followed the same seasonal pattern in normotherms and heterotherms in our model, the energy savings were thus not a result of differences in the heat increment of feeding, but rather lowered metabolic activity at lower $T_{B}$. This metabolic depression resulted in a mid‐winter drop of 5% in daily energy expenditure, or FMR, in heterotherms relative to normotherms. The cumulative benefits of these energy savings throughout the food‐restricted winter months were substantial in our model, allowing females to enter spring and summer with almost 15% greater energy reserves. Since energy reserves, namely body fat, is a strong predictor of reproductive performance in northern mammals including muskoxen (Adamczewski et al., 1998; White et al., 1997), our model predictions clearly highlight and quantify the potential fitness benefits associated with seasonal heterothermy as a mechanism of metabolic energy conservation. Controlled feeding studies in large mammals have shown that lowered metabolic rate and $T_{B}$ can indeed provide significant energy savings and limit body mass loss (e.g., Choshniak et al., 1995; Ostrowski et al., 2006; Turbill et al., 2011), though it is difficult to disentangle the role of metabolic depression from reduced feeding in these studies. Our modeling framework complements field studies and offers unique opportunities to explore different aspects of heterothermy.

### 4.2 Implications for fitness: survival and reproduction

The most likely fitness benefits of heterothermy in mammals experiencing unpredictable and seasonal environments are increased survival and future reproductive performance due to preserved energy reserves (Humphries et al., 2003). These were indeed predicted in our physiological and environmental simulation scenarios. We show how seasonal heterothermy can, to a certain extent, buffer the negative consequences of poor body condition prior to winter (e.g., poor summer forage or effect of nursing a calf in summer) or increased winter food deprivation (e.g., increased snowfall) on winter mortality and energy reserves. To better understand these benefits in terms of reproductive performance, we also modeled the potential impacts of heterothermy during pregnancy. The overwinter energy savings associated with lowered metabolic rate equated to 25% greater energy stores for reproduction at time of spring calving, equivalent to approx. 175 MJ. Put in an ecological context, lactation costs for muskoxen are typically above 5–10 MJ/d (Parker et al., 1990), underlining the significance of the estimated energy saved from heterothermy, or alternatively, the energetic cost to the mother for maintaining normothermic $T_{B}$. This substantial cost to adult females potentially contributes to skipped breeding years, common in free‐ranging populations of muskox and other ungulates (Gaillard et al., 1998; Reynolds, 2001; Schmidt et al., 2015).

Our simulations further showed that heterothermy could increase survival of the mother and its calf when body condition or food restriction becomes increasingly worse. In contrast, the benefit of normothermy during pregnancy was clearly evident as improved birthweight in offspring, which has been shown to have important lifelong fitness consequences (Forchhammer et al., 2001; Harrison et al., 2011; Inchausti & Ginzburg, 2009; Lindström, 1999). This normothermic benefit was only evident if embryo development suffered metabolic depression similar to that observed in the heterothermic mother, while if fetal normothermy is conserved despite metabolic and $T_{B}$ fluctuations in the mother (e.g., Laburn et al., 2002), offspring fitness was not affected by maternal heterothermy. While heterothermy was not observed in pregnant muskox (Schmidt et al., 2020), gestation in many other species occurs simultaneously with fluctuations in $T_{B}$ (see review by McAllan & Geiser, 2014). Thus, while only hypothetical for muskoxen, such a modeling exercise provides meaningful insight for other species.

### 4.3 Heterothermy benefits depend on feeding rates

The benefits of seasonal heterothermy were dependent on whether the mechanisms determining feeding rate were concurrently downregulated with body temperature or not. Since the impacts of heterothermy on feeding rate remain largely unknown for many species, we include model simulations for both possible scenarios, that is, feeding rate is dependent on $T_{B}$ (Arrhenius or $Q_{10}$ effect) or not. If independent of temperature, individuals maximize food intake during periods of scarce resources and the benefits of heterothermy are doubled; relatively high food intake coupled with energy savings from metabolic depression. If feeding depended on $T_{B}$, seasonal heterothermy reduces energy assimilation rates proportional to metabolic depression, largely balancing the metabolic energy savings with reduced food intake.

Most controlled and semi‐controlled studies in ungulates have demonstrated some level of voluntary reduced feed intake in winter (reviewed by Kuntz et al. (2006)), though not all (e.g., Brinkmann et al., 2012). Combined metabolic and feeding studies have shown that metabolic rate and its proxies—body temperature and heart rate—change seasonally independent of food intake (Arnold et al., 2006; Signer et al., 2011; Turbill et al., 2011). For example, Shetland ponies did not show any seasonal shift in food intake despite displaying typical seasonal patterns of heterothermy (Brinkmann et al., 2012). While studies in fat‐storing hibernators have shown how the size and performance of the gut can be significantly reduced (Carey, 1995), muskoxen seem to conserve large digestive capacity across seasons despite changes in microbial community and gut chemistry (Barboza et al., 2006). Altogether, this suggests that observed changes in feeding rate are unlikely to be driven primarily by seasonal fluctuations in $T_{B}$, and more likely driven by environmental or endogenous cues.

### 4.4 Costs of heterothermy

While the adaptive benefits of seasonal heterothermy for survival and future reproduction are clear and substantial, there are likely to be costs associated with abandonment of normothermy. From a theoretical perspective, strict normothermy should allow biochemical processes to perform maximally. Thus, a change in body temperature away from the thermal optimum may reduce performance and push animals closer to thermal thresholds where pathological consequences may ensue (Angilletta et al., 2010; Boyles et al., 2011). However, evidence of this on whole organism performance in heterothermic species is sparse (Boyles et al., 2011). Physiological costs have been well documented in species adopting deep torpor and include oxidative stress, reduced immunocompetence, and neural damage (e.g., reviewed by Humphries et al., 2003), through a myriad of suppressed molecular processes (Carey et al., 2003; Guppy & Withers, 1999). It remains unclear to what extent the relatively small changes in $T_{B}$ documented in muskoxen and other ungulates would induce meaningful physiological costs. In both wild rabbits (*Oryctolagus cuniculus*) and eastern chipmunks (*Tamias striatus*), heterothermy has been associated to a critical trade‐off between survival and reproduction, such that heterothermy increased adult survival at the expense of future reproductive output (Dammhahn et al., 2017; Maloney et al., 2017). In light of the costs and benefits of heterothermy, it must be considered as an adaptive process where optimal thermoregulation is a dynamic function of the organism and its environment (Angilletta et al., 2010; Boyles et al., 2011).

### 4.5 Model caveats and confounding factors

Process‐based modeling approaches such as the one used here provide a powerful and flexible means to investigate the interaction of wildlife with their environment. The strength and flexibility stem partly from the ability to control for confounding biological factors in the model design and thus to isolate the process of interest. In our model, we assumed that the only parameter that changed between heterotherms and normotherms was body temperature, thus enabling us to quantify the effects of temperature‐dependent changes in metabolism overwinter in muskoxen. In the Greenland dataset, heterothermic nonpregnant muskoxen entered the winter season with slightly greater body mass than normothermic pregnant muskoxen (Schmidt et al., 2020), though no obvious differences in body condition were observed (N.M. Schmidt, Pers. Comm.). Similar body condition but different total body mass would suggest differences in structural mass (i.e., fat‐free mass), which would result in additional somatic maintenance costs (∝ *L* ^3^) for the heterothermic muskoxen. Larger animals also have a greater reserve capacity, possibly balancing the additional maintenance costs. We chose to ignore the confounding effect of initial body mass between groups in our modeling approach so as to better understand and explore the direct effect of $T_{B}$ on energetics and fitness, rather than replicate the exact details reported for the wild muskoxen, which had high interindividual variability. We argue this approach provides novel meaningful insights into the costs of thermoregulation in mammals.

Many additional factors not included in this study are known to influence metabolic rate and energy expenditure. Importantly, individual energy reserves likely act as an endogenous signal to trigger energy conservation through heterothermy and hypometabolism (Humphries et al., 2003). Thus, energy reserves not only provide a source of energy during food scarce periods, but are also likely an integral part of the mechanism driving individual variability in heterothermy. Daily activity level can also substantially affect energy expenditure, and animals might change their activity level according to exogenous (e.g., food level, environmental conditions) or endogenous (e.g., energy reserves, hormones) cues (Arnold et al., 2006; Riek et al., 2017). Schmidt et al. (2020) noted minor differences in overwinter activity levels between heterotherms and normotherms, but concluded that the resulting differences in energy expenditure were likely marginal, and thus, this factor was ignored in this study. Knowing the importance of these factors in other wildlife studies, future modeling work could target these explicitly to explore their influence on individual fitness and population dynamics.

Another important aspect of free‐ranging wildlife ecology is animal movement and distribution across the landscape in terms of resource acquisition. For example, individual success in finding local forage patches during snow covered winter months likely contributes to the survival of Greenland muskox (Chimienti et al., 2020). Our model assumes area‐averaged daily snow depth and associated forage accessibility as we simply do not have individual specific environmental data, but this presents a relevant and novel avenue for future research.

## 5 CONCLUSION

Our model uses a well‐established theory of metabolic organization to estimate the energetic dynamics of relative seasonal normothermy and heterothermy in a large mammal. While our model is parameterized for muskoxen, the processes are universal and our framework is therefore applicable to other mammals and environments to test possible effects of changing endogenous and environmental conditions on energetics and fitness. Winter conditions in the Arctic are predicted to become more variable, though tending toward greater precipitation (Krasting et al., 2013). We show that variable harsh conditions (i.e., increased winter snow in our example), and the concomitant reduced forage availability, not only impacts body mass and energy reserves, but also important vital demographic rates like survival and reproduction, hence likely modulating population dynamics. Reduced energy expenditure via heterothermy was predicted to largely curb these negative consequences in low and moderate snow scenarios, suggesting the benefits of this strategy may extend to survival and recruitment in more severe environmental conditions.

## CONFLICT OF INTEREST

We declare no competing interests.

## AUTHOR CONTRIBUTIONS

**Jean‐Pierre Desforges:** Conceptualization (equal); formal analysis (lead); investigation (equal); methodology (lead); project administration (lead); visualization (lead); writing‐original draft (lead); writing‐review & editing (lead). **Floris M. van Beest:** Conceptualization (equal); formal analysis (supporting); funding acquisition (lead); investigation (equal); methodology (equal); project administration (supporting); supervision (equal); validation (supporting); visualization (supporting); writing‐original draft (supporting); writing‐review & editing (supporting). **Gonçalo M. Marques:** Formal analysis (supporting); methodology (supporting); supervision (supporting); validation (supporting); visualization (supporting); writing‐review & editing (equal). **Stine H. Pedersen:** Data curation (equal); methodology (supporting); resources (supporting); validation (supporting); visualization (supporting); writing‐original draft (supporting); writing‐review & editing (supporting). **Larissa T. Beumer:** Formal analysis (supporting); methodology (supporting); resources (supporting); visualization (supporting); writing‐original draft (supporting); writing‐review & editing (supporting). **Marianna Chimienti:** Formal analysis (supporting); methodology (supporting); resources (supporting); visualization (supporting); writing‐original draft (supporting); writing‐review & editing (supporting). **Niels Martin Schmidt:** Conceptualization (equal); data curation (lead); formal analysis (supporting); funding acquisition (supporting); investigation (equal); methodology (equal); project administration (supporting); resources (supporting); supervision (equal); validation (supporting); visualization (supporting); writing‐original draft (supporting); writing‐review & editing (supporting).

## Supporting information

Appendix S1

## Data Availability

Full model description and model code are provided in Appendix S1. Supporting data on muskoxen body temperature can be found at https://doi.org/10.5281/zenodo.3584998.

## References

[ece37049-bib-0001] Adamczewski, J. , Fargey, P. J. , Laarveld, B. , Gunn, A. , & Flood, P. F. (1998). The influence of fatness on the likelihood of early‐winter pregnancy in muskoxen. Theriogenology, 50, 605–614. 10.1016/S0093-691X(98)00165-4 10732151

[ece37049-bib-0002] Adamczewski, J. , Flood, P. F. , & Gunn, A. (1997). Seasonal patterns in body composition and reproduction of female muskoxen (*Ovibos moschatus*). Journal of Zoology, 241, 245–269.

[ece37049-bib-0003] Adamczewski, J. , Gunn, Å. , Laarveld, B. , & Flood, P. F. (1992). Seasonal changes in weight, condition and nutrition of free‐ranging and captive muskox females. Rangifer, 12, 179–183. 10.7557/2.12.3.1041

[ece37049-bib-0004] Angilletta, M. , Cooper, B. , Schuler, M. , & Boyles, J. (2010). The evolution of thermal physiology in endotherms. Frontiers in Bioscience, E2, 861–881. 10.2741/e148 20515760

[ece37049-bib-0005] Arnold, W. , Ruf, T. , & Kuntz, R. (2006). Seasonal adjustment of energy budget in a large wild mammal, the Przewalski horse (*Equus ferus przewalskii*) II. Energy expenditure. Journal of Experimental Biology, 209, 4566–4573. 10.1242/jeb.02536 17079726

[ece37049-bib-0006] Arnold, W. , Ruf, T. , Loe, L. E. , Irvine, R. J. , Ropstad, E. , Veiberg, V. , & Albon, S. D. (2018). Circadian rhythmicity persists through the Polar night and midnight sun in Svalbard reindeer. Scientific Reports, 8, 1–12. 10.1038/s41598-018-32778-4 30262810 PMC6160466

[ece37049-bib-0007] Arnold, W. , Ruf, T. , Reimoser, S. , Tataruch, F. , Onderscheka, K. , & Schober, F. (2004). Nocturnal hypometabolism as an overwintering strategy of red deer (*Cervus elaphus*). American Journal of Physiology‐Regulatory, Integrative and Comparative Physiology, 286, R174–R181. 10.1152/ajpregu.00593.2002 12969877

[ece37049-bib-0008] Barboza, P. S. , Peltier, T. C. , & Forster, R. J. (2006). Ruminal fermentation and fill change with season in an Arctic grazer: Responses to hyperphagia and hypophagia in muskoxen (*Ovibos moschatus*). Physiological and Biochemical Zoology, 79, 497–513. 16691516 10.1086/501058

[ece37049-bib-0009] Boyles, J. G. , Seebacher, F. , Smit, B. , & McKechnie, A. E. (2011). Adaptive thermoregulation in endotherms may alter responses to climate change. Integrative and Comparative Biology, 51, 676–690. 10.1093/icb/icr053 21690108

[ece37049-bib-0010] Boyles, J. G. , Thompson, A. B. , McKechnie, A. E. , Malan, E. , Humphries, M. M. , & Careau, V. (2013). A global heterothermic continuum in mammals. Global Ecology and Biogeography, 22, 1029–1039. 10.1111/geb.12077

[ece37049-bib-0011] Brinkmann, L. , Gerken, M. , & Riek, A. (2012). Adaptation strategies to seasonal changes in environmental conditions of a domesticated horse breed, the Shetland pony (*Equus ferus caballus*). Journal of Experimental Biology, 215, 1061–1068. 10.1242/jeb.064832 22399650

[ece37049-bib-0012] Bronson, F. H. (2009). Climate change and seasonal reproduction in mammals. Philosophical Transactions of the Royal Society B: Biological Sciences, 364, 3331–3340. 10.1098/rstb.2009.0140 PMC2781850 19833645

[ece37049-bib-0013] Carey, H. (1995). Gut feelings about hibernation. Physiology, 10, 55–61. 10.1152/physiologyonline.1995.10.2.55

[ece37049-bib-0014] Carey, H. V. , Andrews, M. T. , & Martin, S. L. (2003). Mammalian hibernation: Cellular and molecular responses to depressed metabolism and low temperature. Physiological Reviews, 83, 1153–1181. 10.1152/physrev.00008.2003 14506303

[ece37049-bib-0015] Chimienti, M. , Desforges, J. , Beumer, L. T. , Nabe‐nielsen, J. , Van Beest, F. M. , & Martin, N. (2020). Energetics as common currency for integrating high resolution activity patterns into dynamic energy budget‐individual based models. Ecological Modelling, 434, 109250 10.1016/j.ecolmodel.2020.109250

[ece37049-bib-0016] Choshniak, I. , Ben‐Kohav, N. , Taylor, C. R. , Robertshaw, D. , Barnes, R. J. , Dobson, A. , Belkin, V. , & Shkolnik, A. (1995). Metabolic adaptations for desert survival in the Bedouin goat. American Journal of Physiology, 268, R1101–R1110. 10.1152/ajpregu.1995.268.5.R1101 7771568

[ece37049-bib-0017] Coulson, T. (2001). Age, sex, density, winter weather, and population crashes in soay sheep. Science, 292, 1528–1531. 10.1126/science.292.5521.1528 11375487

[ece37049-bib-0018] Coulson, T. , Milner‐Gulland, E. J. , & Clutton‐Brock, T. (2000). The relative roles of density and climatic variation on population dynamics and fecundity rates in three contrasting ungulate species. Proceedings of the Royal Society B: Biological Sciences, 267, 1771–1779. 10.1098/rspb.2000.1209 PMC1690729 12233776

[ece37049-bib-0019] Dammhahn, M. , Landry‐Cuerrier, M. , Réale, D. , Garant, D. , & Humphries, M. M. (2017). Individual variation in energy‐saving heterothermy affects survival and reproductive success. Functional Ecology, 31, 866–875. 10.1111/1365-2435.12797

[ece37049-bib-0020] Dehnel, A. (1949). Studies on the genus Sorex L. Annales Universitatis Mariae Curie‐Sklodowska, Sectio C, 4, 17–102. 24538596

[ece37049-bib-0021] Desforges, J.‐P. , Marques, G. M. , Beumer, L. T. , Chimienti, M. , Blake, J. , Rowell, J. E. , Adamczewski, J. , Schmidt, N. M. , & van Beest, F. M. (2019). Quantification of the full lifecycle bioenergetics of a large mammal in the high Arctic. Ecological Modelling, 401, 27–39. 10.1016/j.ecolmodel.2019.03.013

[ece37049-bib-0022] Forchhammer, M. C. , Clutton‐Brock, T. H. , Lindström, J. , & Albon, S. D. (2001). Climate and population density induce long‐term cohort variation in a northern ungulate. Journal of Animal Ecology, 70, 721–729. 10.1046/j.0021-8790.2001.00532.x

[ece37049-bib-0023] Gaillard, J. M. , Festa‐Bianchet, M. , & Yoccoz, N. G. (1998). Population dynamics of large herbivores: Variable recruitment with constant adult survival. Trends in Ecology and Evolution, 13, 58–63. 10.1016/S0169-5347(97)01237-8 21238201

[ece37049-bib-0024] Gaillard, J.‐M. , Festa‐Bianchet, M. , Yoccoz, N. G. , Loison, A. , & Toïgo, C. (2000). Temporal variation in fitness components and population dynamics of large herbivores. Annual Review of Ecology and Systematics, 31, 367–393. 10.1146/annurev.ecolsys.31.1.367

[ece37049-bib-0025] Geiser, F. (2004). Metabolic rate and body temperature reduction during hibernation and daily torpor. Annual Review of Physiology, 66, 239–274. 10.1146/annurev.physiol.66.032102.115105 14977403

[ece37049-bib-0026] Guppy, M. , & Withers, P. (1999). Metabolic depression in animals: Physiological perspectives and biochemical generalizations. Biological Reviews, 74, 1–40. 10.1017/S0006323198005258 10396183

[ece37049-bib-0027] Hansen, B. U. , Sigsgaard, C. , Rasmussen, L. , Cappelen, J. , Hinkler, J. , Mernild, S. H. , Petersen, D. , Tamstorf, M. P. , Rasch, M. , & Hasholt, B. (2008). Present‐day climate at Zackenberg. Advances in Ecological Research, 40, 111–149.

[ece37049-bib-0028] Harrison, X. A. , Blount, J. D. , Inger, R. , Norris, D. R. , & Bearhop, S. (2011). Carry‐over effects as drivers of fitness differences in animals. Journal of Animal Ecology, 80, 4–18. 10.1111/j.1365-2656.2010.01740.x 20726924

[ece37049-bib-0029] Heldmaier, G. , Ortmann, S. , & Elvert, R. (2004). Natural hypometabolism during hibernation and daily torpor in mammals. Respiratory Physiology and Neurobiology, 141, 317–329. 10.1016/j.resp.2004.03.014 15288602

[ece37049-bib-0030] Helle, T. , & Kojola, I. (2008). Demographics in an alpine reindeer herd: Effects of density and winter weather. Ecography, 31, 221–230. 10.1111/j.0906-7590.2008.4912.x

[ece37049-bib-0031] Humphries, M. M. , Thomas, D. W. , & Kramer, D. L. (2003). The role of energy availability in mammalian hibernation: A cost‐benefit approach. Physiological and Biochemical Zoology, 76, 165–179. 10.1086/367950 12794670

[ece37049-bib-0032] Inchausti, P. , & Ginzburg, L. R. (2009). Maternal effects mechanism of population cycling: A formidable competitor to the traditional predator‐prey view. Philosophical Transactions of the Royal Society B: Biological Sciences, 364, 1117–1124. 10.1098/rstb.2008.0292 PMC2666685 19324616

[ece37049-bib-0033] Kooijman, S. (2010). Dynamic Energy Budget theory for metabolic organisation (3rd ed.). Cambridge University Press. 10.1111/j.1469-185X.2006.00006.x 17313526

[ece37049-bib-0034] Krasting, J. P. , Broccoli, A. J. , Dixon, K. W. , & Lanzante, J. R. (2013). Future changes in Northern Hemisphere snowfall. Journal of Climate, 26, 7813–7828. 10.1175/JCLI-D-12-00832.1

[ece37049-bib-0035] Kuntz, R. , Kubalek, C. , Ruf, T. , Tataruch, F. , & Arnold, W. (2006). Seasonal adjustment of energy budget in a large wild mammal, the Przewalski horse (*Equus ferus przewalskii*) II. Energy expenditure. Journal of Experimental Biology, 209, 4566–4573. 10.1242/jeb.02535 17079725

[ece37049-bib-0036] Kutz, S. , Rowell, J. , Adamczewski, J. , Gunn, A. , Cuyler, C. , Aleuy, O. A. , Austin, M. , Berger, J. , Blake, J. , Bondo, K. , Dalton, C. , Dobson, A. , Di Francesco, J. , Gerlach, C. , Kafle, P. , Mavrot, F. , Mosbacher, J. , Murray, M. , Nascou, A. , … Ytrehus, B. (2017). Muskox health ecology symposium 2016: Gathering to share knowledge on Umingmak in a time of rapid change. Arctic, 70, 225–236. 10.14430/arctic4656

[ece37049-bib-0037] Laburn, H. P. , Faurie, A. , Goelst, K. , & Mitchell, D. (2002). Effects on fetal and maternal body temperatures of exposure of pregnant ewes to heat, cold, and exercise. Journal of Applied Physiology, 92, 802–808. 10.1152/japplphysiol.00109.2001 11796695

[ece37049-bib-0038] Lawler, J. P. , & White, R. G. (1997). Seasonal changes in metabolic rates in muskoxen following twenty‐ four hours of starvation. Rangifer, 17, 135–138. 10.7557/2.17.3.1365

[ece37049-bib-0039] Lindström, J. (1999). Early development and fitness in birds and mammals. Trends in Ecology & Evolution, 14, 343–348. 10.1016/S0169-5347(99)01639-0 10441307

[ece37049-bib-0040] Liston, G. E. , & Elder, K. (2006a). A meteorological distribution system for high‐resolution terrestrial modeling (MicroMet). Journal of Hydrometeorology, 7, 217–234. 10.1175/JHM486.1

[ece37049-bib-0041] Liston, G. E. , & Elder, K. (2006b). A distributed snow‐evolution modeling system (SnowModel). Journal of Hydrometeorology, 7, 1259–1276. 10.1175/JHM548.1

[ece37049-bib-0042] Maloney, S. K. , Marsh, M. K. , McLeod, S. R. , & Fuller, A. (2017). Heterothermy is associated with reduced fitness in wild rabbits. Biology Letters, 13, 20170521 10.1098/rsbl.2017.0521 29212751 PMC5746534

[ece37049-bib-0043] McAllan, B. M. , & Geiser, F. (2014). Torpor during reproduction in mammals and birds: Dealing with an energetic conundrum. Integrative and Comparative Biology, 54, 516–532. 10.1093/icb/icu093 24973362

[ece37049-bib-0044] McKechnie, A. E. , & Mzilikazi, N. (2011). Heterothermy in afrotropical mammals and birds: A review. Integrative and Comparative Biology, 51, 349–363. 10.1093/icb/icr035 21705792

[ece37049-bib-0045] Munn, A. J. , Barboza, P. S. , & Dehn, J. (2009). Sensible heat loss from muskoxen (*Ovibos moschatus*) feeding in winter: Small calves are not at a thermal disadvantage compared with adult cows. Physiological and Biochemical Zoology, 82, 455–467. 19569931 10.1086/605400

[ece37049-bib-0046] Nowack, J. , Stawski, C. , & Geiser, F. (2017). More functions of torpor and their roles in a changing world. Journal of Comparative Physiology B: Biochemical, Systemic, and Environmental Physiology, 187, 889–897. 10.1007/s00360-017-1100-y PMC5486538 28432393

[ece37049-bib-0047] Ostrowski, S. , William, J. , Mesochina, P. , & Sauerwein, H. (2006). Physiological acclimation of a desert antelope, Arabian oryx (*Oryx leucoryx*), to long‐term food and water restriction. Journal of Comparative Physiology B, 176, 191–201. 10.1007/s00360-005-0040-0 16283332

[ece37049-bib-0048] Parker, K. L. , White, R. G. , Gillingham, M. P. , & Holleman, D. F. (1990). Comparison of energy metabolism in relation to daily activity and milk consumption by caribou and muskox neonates. Canadian Journal of Zoology, 68, 106–114. 10.1139/z90-015

[ece37049-bib-0049] Pedersen, S. H. , Liston, G. E. , Tamstorf, M. P. , Abermann, J. , Lund, M. , & Schmidt, N. M. (2018). Quantifying snow controls on vegetation greenness. Ecosphere, 9, e02309 10.1002/ecs2.2309

[ece37049-bib-0050] R core Team (2020). R: A language and environment for statistical computing. R Foundation for Statistical Computing.

[ece37049-bib-0051] Reynolds, P. E. (2001). Reproductive patterns of female muskoxen in northeastern Alaska. Alces, 37, 403–410.

[ece37049-bib-0052] Riek, A. , Brinkmann, L. , Gauly, M. , Perica, J. , Ruf, T. , Arnold, W. , Hambly, C. , Speakman, J. R. , & Gerken, M. (2017). Seasonal changes in energy expenditure, body temperature and activity patterns in llamas (*Lama glama*). Scientific Reports, 7, 7600. 28790450 10.1038/s41598-017-07946-7 PMC5548813

[ece37049-bib-0053] Riek, A. , Stölzl, A. , Marquina Bernedo, R. , Ruf, T. , Arnold, W. , Hambly, C. , Speakman, J. R. , & Gerken, M. (2019). Energy expenditure and body temperature variations in llamas living in the High Andes of Peru. Scientific Reports, 9, 1–11. 10.1038/s41598-019-40576-9 30858417 PMC6411917

[ece37049-bib-0054] Ruf, T. , & Geiser, F. (2015). Daily torpor and hibernation in birds and mammals. Biological Reviews, 90, 891–926. 10.1111/brv.12137 25123049 PMC4351926

[ece37049-bib-0055] Schmidt, N. M. , van Beest, F. M. , Mosbacher, J. B. , Stelvig, M. , Hansen, L. H. , Nabe‐Nielsen, J. , & Grøndahl, C. (2016). Ungulate movement in an extreme seasonal environment: year‐round movement patterns of high‐arctic muskoxen. Wildlife Biology, 22(6), 253–267. 10.2981/wlb.00219

[ece37049-bib-0056] Schmidt, N. M. , Grøndahl, C. , Evans, A. L. , Desforges, J.‐P. , Blake, J. , Hansen, L. H. , Beumer, L. T. , Mosbacher, J. B. , Stelvig, M. , Greunz, E. M. , Chimienti, M. , & van Beest, F. M. (2020). On the interplay between hypothermia and reproduction in a high arctic ungulate. Scientific Reports, 10, 1514 10.1038/s41598-020-58298-8 32001737 PMC6992616

[ece37049-bib-0057] Schmidt, N. M. , Pedersen, S. H. , Mosbacher, J. B. , & Hansen, L. H. (2015). Long‐term patterns of muskox (*Ovibos moschatus*) demographics in high arctic Greenland. Polar Biology, 38, 1667–1675. 10.1007/s00300-015-1733-9

[ece37049-bib-0058] Signer, C. , Ruf, T. , & Arnold, W. (2011). Hypometabolism and basking: The strategies of Alpine ibex to endure harsh over‐wintering conditions. Functional Ecology, 25, 537–547. 10.1111/j.1365-2435.2010.01806.x

[ece37049-bib-0059] Stillman, R. A. , Railsback, S. F. , Giske, J. , Berger, U. , & Grimm, V. (2015). Making predictions in a changing world: The benefits of individual‐based ecology. BioScience, 65, 140–150. 10.1093/biosci/biu192 26955076 PMC4778170

[ece37049-bib-0060] Thiel, A. , Evans, A. L. , Fuchs, B. , Arnemo, J. M. , Aronsson, M. , & Persson, J. (2019). Effects of reproduction and environmental factors on body temperature and activity patterns of wolverines. Frontiers in Zoology, 16, 1–11. 10.1186/s12983-019-0319-8 31236127 PMC6580505

[ece37049-bib-0061] Turbill, C. , Ruf, T. , Mang, T. , & Arnold, W. (2011). Regulation of heart rate and rumen temperature in red deer: Effects of season and food intake. Journal of Experimental Biology, 214, 963–970. 10.1242/jeb.052282 PMC3280896 21346124

[ece37049-bib-0062] White, R. , Rowell, J. , & Hauer, W. (1997). The role of nutrition, body condition and lactation on calving success in muskoxen. Journal of Zoology, 243, 13–20. 10.1111/j.1469-7998.1997.tb05752.x

