# Supplementary material for: Quantifying energetic and fitness consequences of seasonal heterothermy in an Arctic ungulate
Source: Ecol Evol. 2020 Nov 22;11(1):338–51. doi: 10.1002/ece3.7049 (PMC7790657; doi:10.1002/ece3.7049)
Supplement: Supplementary file 1 — Appendix S1 [file ECE3-11-338-s001.docx]

**Appendix I**

*This is an ODD (“Overview, Design concepts, and Details”) document following the protocol suggested by**Grimm et al., 2006,**Grimm et al., 2010 for our model presented in*

**Quantifying energetic and fitness consequences of seasonal heterothermy in an Arctic ungulate**

Table of Contents

[1 Problem Formulation 3](#_Toc38278891)

[2 Model Description 3](#_Toc38278892)

[2.1 Purpose 4](#_Toc38278895)

[2.2 Entities, State Variables, and Scales 5](#_Toc38278896)

[2.3 Parameter Estimation 6](#_Toc38278897)

[2.4 Process Overview and Scheduling 6](#_Toc38278898)

[2.5 Design Concepts 7](#_Toc38278899)

[2.6 Initialization 9](#_Toc38278900)

[2.7 Input Data 9](#_Toc38278901)

[2.8 Sub-models 11](#_Toc38278902)

[3 Sensitivity Analysis 20](#_Toc38278903)

[4 Model Scenarios 21](#_Toc38278904)

[4.1 Scenario 1 21](#_Toc38278907)

[4.2 Scenario 2 21](#_Toc38278908)

[4.3 Scenario 3 21](#_Toc38278909)

[4.4 Temperature dependent feeding scenarios 22](#_Toc38278910)

[5 References 23](#_Toc38278911)

# Problem Formulation

The muskox (*Ovibos moschatus*) is one of the few large-bodied herbivores in the circumpolar Arctic and as such plays an important role in the structure and function of tundra ecosystems (Falk et al., 2015; Mosbacher et al., 2018). As a year-round Arctic resident, muskoxen experience some of the most extreme seasonal fluctuations in climatic and environmental conditions in the world, resulting in marked temporal variation in the availability and quality of their food base (Mosbacher et al., 2016). Seasonal changes in external conditions, especially ambient temperature, have important consequences for endotherms as they need to maintain high body temperatures for optimal physiological function. Indeed, temperature regulation can be energetically costly, especially during stressful periods of cold temperatures and resource scarcity (Geiser, 2004; Ruf and Geiser, 2015). For endothermic species that are unable to migrate, such as the muskoxen, behavioral and physiological adaptations are the only available strategies to minimize exposure to harsh conditions and to mitigate the impacts of energy limitation. Besides using local refuges (Signer et al., 2011) species can reduce their energy expenditure by adjusting locomotor activity, body temperature, and metabolic rates (Arnold et al., 2006; Brinkmann et al., 2012; Riek et al., 2017). Reduced body temperature and metabolic rate associated with hypometabolism can range from long term hibernation to daily torpor (Geiser, 2004).

Winter snow conditions have been shown to be a major determinant of northern ungulate body condition, reproduction, and population dynamics (Gaillard et al., 2000; Helle and Kojola, 2008; Schmidt et al., 2015), highlighting the impact of seasonal environments on animal fitness. Seasonal heterothermy and hypometabolism have been demonstrated for several ungulate species in response to adverse environmental conditions (Brinkmann et al., 2012; Riek et al., 2017; Signer et al., 2011; Turbill et al., 2011). For capital breeding species like the muskox, energy reserves are also used in winter to cover costly reproductive needs associated with gestation and lactation (Adamczewski et al., 1997). A recent study on muskoxen in East Greenland found clear signs of over-winter heterothermy, but this strategy was restricted to non-pregnant females as pregnant females maintained a stable temperature profile throughout the winter months (Schmidt et al., *2020*).

The model presented here builds on our previous muskox energetics model (Desforges et al., 2019) to incorporate the influence of environmental data on forage acquisition and body temperature on rates of energy uptake and usage.

# Model Description

The model description follows the ODD (Overview, Design concepts and Details) protocol for describing individual-based models (Grimm et al., 2006). The DEB-IBM was developed in the open-source agent-based modelling framework NetLogo (version 6.0.2, August 4 2017). We follow standard DEB notations for parameters (Kooijman, 2010); a summary of model parameters can be found in Table 1 and a model schematic in Figure 1.


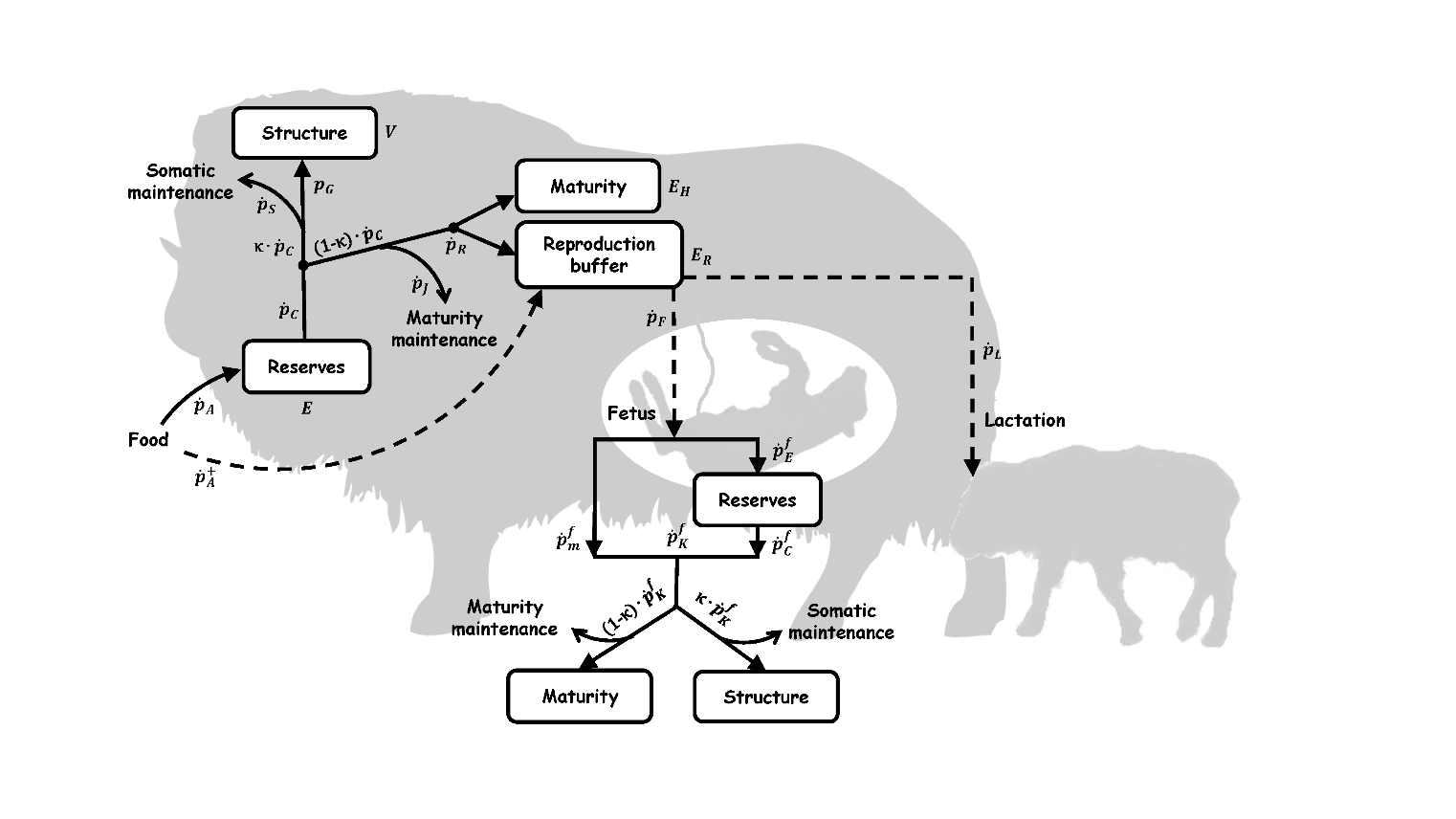


Figure 1. Schematic representation of the generic muskox dynamic energy budget (DEB) model. Lactation was not included in the current implementation of the model. Figure taken from Desforges et al. (2019).


## Purpose

The purpose of this model is to simulate the overwinter energetics and fitness consequences of heterothermy in high Arctic muskox using a dynamic energy budget linked individual based model (DEB-IBM). This model builds on the DEB-IBM framework previously published for muskoxen in Desforges et al. (2019) by incorporating environmental data and temperature correction on energetics for different body temperatures associated with heterothermy.

Table 1. Model parameters. Value determined during parameter estimation unless otherwise stated.

|  | **Symbol** | **Unit** | **Value** |
| --- | --- | --- | --- |
| ***Feeding*** |  |  |  |
| maximum specific assimilation rate | $\{\dot{p}_{Am}\}$ | J d^-1^ cm^-2^ | 14557.34 |
| scaled functional feeding response | $f$ | -- | function of snow depth |
| snow depth threshold for functional response | $\mathrm{SD}_{thresh}$ | -- | 0.70 |
| digestion efficiency | $\kappa_{X}$ | -- | 0.30 |
| ***Energetics*** |  |  |  |
| energy conductance | $\dot{v}$ | cm d^-1^ | 0.33 |
| allocation fraction to soma | $\kappa$ | -- | 0.9779 |
| volume-specific somatic maintenance rate | $[\dot{p}_{M}]$ | J d^-1^ cm^-3^ | 434.01 |
| maturity maintenance rate constant | $k_{J}$ | d^-1^ | 1.23 x10^-7^ |
| specific cost for structure | $[E_{G}]$ | J cm^-3^ | 7835 |
| maturity at birth | $E_{H}^{b}$ | J | 7.10 x10^5^ |
| ***Temperature Correction*** |  |  |  |
| Arrhenius temperature | $T_{A}$ | K | 8000^a^ |
| reference temperature | $T_{REF}$ | K | 293.15 |
| ***Reproduction*** |  |  |  |
| reproduction efficiency | $\kappa_{R}$ | -- | 0.95^a^ |
| embryonic arrest period | $t_{0}$ | d | 85 |
| initial structural length of fetus | $L_{0}$ | cm | 0.0001 |
| initial reserves of fetus | $E_{0}$ | J | function of reserve density of the mother |
| logistic fetal survival effect coefficient | $f\_surv\_coef$ | -- | 0.625 |
| logistic fetal survival steepness coefficient | $k_{i}$ | -- | 35 |
| ***Mortality*** |  |  |  |
| adult logistic starvation survival effect coefficient | $starve\_coef$ | -- | 0.15 |
| logistic starvation survival steepness coefficient | $k_{s}$ | -- | 30 |
| scaled total reserve density death threshold | $e_{death}$ | -- | 0.20 |
| ***Conversions*** |  |  |  |
| specific density of structure and reserves | $dV$ | g cm^-3^ | 0.3^a^ |
| chemical potential of reserve | $\mu_{E}$ | J mol^-1^ | 550000^a^ |
| chemical potential of structure | $\mu_{V}$ | J mol^-1^ | 500000^a^ |
| molecular weight of reserve | $\omega_{E}$ | g mol^-1^ | 23.9^a^ |

^a^ Standard value from Kooijman (2010).

## Entities, State Variables, and Scales

Entities in the model are individual adult females and their offspring. The current model is non-spatial and time is represented continuously (ordinary differential equations), but conceptually the model is based on daily time steps. The model begins on October 1^st^ (day 1) and runs on a daily time-step to the end of April (day 210), which covers embryonic arrest, active gestation, and birth as well as the period with highest snow accumulations and least amount of forage. For each model run, 25 adult individuals are initialized and their reproductive status is set to either pregnant or non-pregnant. Reserves ($E$) are set at their size-specific maximum by multiplying the species-specific maximum reserve density (${[E}_{m}]$) to structural volume ($L^{3}$). The reproduction buffer was defined as a linear function of reserves according to $E_{R}= 0.0765\cdot E+127990205$, the relationship found from the results of our previous model in wild muskox (Desforges et al., 2019). The model was originally parameterized for large captive muskox (Desforges et al., 2019), and 80% of maximum reserves was used here to match observed body weights and condition of smaller wild muskoxen. We define total scaled reserves ($e_{tot}$) as the sum of reserves and reproduction buffer scaled by the maximum reserve capacity ($E_{m}= {[E}_{m}]\cdot L^{3}$), and use this index as a proxy for body condition in functions that determine probabilities for survival and reproduction.

The environment is updated daily and is composed of mean snow depth estimated across the muskox census area in Zackenberg, Greenland. These data were produced by Pedersen et al. (2018) using MicroMet and SnowModel (Liston and Elder, 2006a, 2006b). To capture the typical snow conditions experienced by muskoxen in this area, we take the average daily values over the past 18 years (2000-2018). Snow depth is used as a proxy for the food availability parameter $f$ (functional response), a dimensionless variable ranging from 0 to 1, that controls to the total flux of energy assimilated into the individuals. See further details in ‘2.7 - Input Data’.

## Parameter Estimation

The software package DEBtool (http://www.bio.vu.nl/thb/deb/deblab/debtool) was used to estimate the DEB-related parameters for the muskox, as described in Desforges et al. (2019). The DEB model was further developed and implemented in an individual based model (IBM) framework in the open-source agent-based modelling program NetLogo (version 6.0.2, August 4 2017). The DEB parameters were estimated using data from a captive research herd of muskoxen held at the Large Animal Research Station run by the University of Alaska Fairbanks. Data include zero-variate (age and weight at birth, weaning, puberty, and asymptotic size, as well as maximum reproductive rate) and univariate (foetal and full-lifetime body weight growth curves, milk production and intake rates, feeding rates, and seasonal body condition) types of information. See our previous ODD model description for details (Desforges et al., 2019).

## Process Overview and Scheduling

Individuals update their DEB state variables every time step by solving a set of differential equations. Discrete events, such as birth and death, may occur based on the outcomes of these equations. Processes within the DEB model mostly follow the standard energy allocation theory as described by Kooijman (2010), and described in detail in the sub-model sections and in Desforges et al. (2019). The model used here is a simplification of our previous model as we were only interested in over-winter dynamics in adult females. We therefore do not include aspects of lactation, maturation, growth, ageing, or upregulated feeding during summer months.

Individuals that are not dependent on others for energy (adults), take in energy that is available in the environment. Daily food availability is a function of snow depth throughout the year such that maximum and minimum feeding occur in summer and winter, respectively, when snow depths are accordingly low and high. Assimilated energy is used by the individual to fuel the physiological processes of maintenance, growth, maturation, and reproduction, as well as maintain reserves. When energy from reserves is insufficient to cover maintenance costs, individuals enter starvation mode to prioritize energy use. Individual mortality can occur via starvation or fetal abortion/mortality. Fetuses are dependent on their mother for energy. Energy is available from the total energy reserves of the mother, which included both the reserves and reproduction buffer, and these dictated whether breeding occurs, the embryo is aborted, and if the mother gives birth.

The effect of body temperature change is implemented using a temperature correction factor ($TC$) applied to DEB rate parameters ($\left[ \dot{p}_{M} \right], \dot{v}, \dot{k}_{J}$) (Kooijman, 2010). We include two possible influences of heterothermy on reproduction: 1) embryo homeostasis is preserved and independent of maternal $T_{B}$, and 2) embryo development is a function of maternal $T_{B}$ and thus metabolic rates in the embryo energy budget are temperature corrected. See further details in ‘2.8 – temperature correction’.

The following pseudo-code describes the scheduling of events within each time step of the model. Sub-models are described in detail in the sub-model section below.

Setup

- define parameter values
- initialize muskox population
  - set pregnancy status
- temperature correction
- load external snow data

All adults

- temperature correction
- calculate reserves
- calculate maturity/reproduction buffer
- calculate growth
- calculate starvation
- calculate mortality
- update variables

All pregnant individuals

- calculate foetal development

## **Design Concepts**

*Emergence*: Individual life-history traits, such as growth (body weight), energy reserves, maturation time (age at birth), reproductive effort, and death, emerge from rules of metabolic organization and driven by the influence of daily differences in food availability and body temperature.

*Interaction*: Interactions in our model occur between mother and offspring. The embryo is linked to its mother via energy and mass transfer, where all required energy in the embryo DEB is derived from the mother.

*Stochasticity*: Stochasticity in the model is introduced for body temperature ($T_{B}$), the maximum specific assimilation rate ($\left\{ \dot{p}_{Am} \right\}$), mortality, and reproductive success.

Individual variability was included for $T_{B}$ and $\left\{ \dot{p}_{Am} \right\}$ using a scatter-multiplier (Martin et al., 2012), thus affecting metabolic rates and food intake, respectively. The scatter-multiplier is a log-normally distributed random number with a selected value for the coefficient of variation (cv) applied to the initial parameter value:

$$scatter-multiplier=e^{random-normal 0 cv}$$

Empirical data of free-ranging adult female muskoxen in high Arctic Greenland revealed a divergent seasonal pattern of $T_{B}$, in which pregnant animals kept strict normothermy (mean 38.2 ^o^C) throughout winter while non-pregnant animals saw a gradual decline in $T_{B}$ over-winter as ambient temperature and snow cover/depth increased (Schmidt et al. 2020). We used the mean daily body temperature of normotherms and heterotherms and implement additional individual variability in the model using the scatter-multiplier. Heterothermic muskoxen were observed to have a greater range of daily $T_{B}$ than normotherms, thus the coefficients of variation were set at 0.15 and 0.10 respectively to arrive at observed levels of variability ($T_{B} x scatter-multiplier)$. The resulting modelled $T_{B}$ patterns are illustrated below (Figure 2).

Figure 2. Daily over-winter body temperature profiles of normotherm and heterotherm adult females. Secondary y-axes display the percentage change from starting values.

The value of cv for the scatter-multiplier applied to $\left\{ \dot{p}_{Am} \right\}$ was set at 2.5% in order to simulate approximate observed variation in ingestion rates and resulting body size (Desforges et al. 2019).

Two aspects of mortality are probabilistic: starvation-related mortality and fetal death/ reproductive success. Reproductive success is probabilistically defined and dependent on the total energy reserves of the mother at the onset of active fetal development (i.e. after the embryo arrest period, $t_{0}$). See related sub-models (section 2.8) for additional details.

*Observation*: Any individual variable of interest can easily be observed and recorded in NetLogo, including detailed energy fluxes for physiological processes (see Desforges et al., 2019) and population metrics. Key outputs for this model implementation are those describing relevant fitness metrics, including body weight, energy reserves, field metabolic rate/daily energy expenditure, ingestion rates, and survival.

## Initialization

For our purpose here, the DEB-IBM targeted only the winter season and the impacts of lowered body temperature. The model begins on October 1^st^ (day 1) and runs on a daily time-step to the end of April (day 210), which covers embryonic arrest, active gestation, and birth as well as the period with highest snow accumulations and least amount of forage. For each model run, 25 adult individuals are initialized and their reproductive status is set to either pregnant or non-pregnant. Reserves ($E$) are set at their size-specific maximum by multiplying the species-specific maximum reserve density (${[E}_{m}]$) to structural volume ($L^{3}$):

$$E= {[E}_{m}] L^{3}$$

where

$${[E}_{m}]=\frac{\left\{ \dot{p}_{Am} \right\}}{\dot{v}}$$

The reproduction buffer was defined as a linear function of reserves according to $E_{R}= 0.0765\cdot E+127990205$, the relationship found from the results of our previous model in wild muskox (Desforges et al., 2019). The model was originally parameterized for large captive muskox (Desforges et al., 2019), and 80% of maximum reserves was used here to match observed body weights and condition of smaller wild muskoxen. We define total scaled reserves ($e_{tot}$) as the sum of reserves and reproduction buffer scaled by the maximum reserve capacity ($E_{m}= {[E}_{m}]\cdot L^{3}$), and use this index as a proxy for body condition in functions that determine probabilities for survival and reproduction.

## Input Data

Time-specific food availability and acquisition in the model is a function of snow depth. Mean daily snow depth across the muskox census area in Zackenberg, Greenland, is used as a proxy for the scaled functional response (*f*), which is a condensed proxy for forage acquisition (e.g. food availability, searching, handling, etc) and takes values between 0 (starvation) and 1 (maximum feeding) (Kooijman, 2010; Marn et al., 2017). As input of snow conditions, we used modeled mean daily snow depth (cm) as a proxy for food availability and acquisition throughout winter. These data were produced over the muskox monitoring area in Zackenberg in Greenland by Pedersen et al. (2018) using MicroMet and SnowModel (Liston and Elder, 2006a, 2006b). Snow depth is given for each 300 m^2^ pixel of the Zackenberg census area, which were averaged to get a single daily value of snow depth. We use the average here because our model is not spatially explicit and we strive to capture the response at the population level within the entire area. To capture the typical snow conditions experienced by muskoxen in this area, we take the average daily values over the past 18 years (2000-2018).

Before the average daily snow depth value could be applied as a proxy for *f* we needed to resolve several scaling issues. First, snow depth was scaled to the maximum value in the dataset in order to derive values consistent with the functional response (i.e. between 0 and 1). Next, we calculate the inverse of the scaled snow depth to give a seasonal pattern that again is consistent with the functional response, meaning higher values equate to greater forage acquisition and vice versa. This was necessary as snow depth is naturally at its highest in winter and lowest in summer, while feeding is the opposite. Using these variables as they are in the model resulted in rapid collapse of the initial muskox population during model testing as assimilation rates were too low to cover the metabolic needs of the individuals. In other words, winter forage accessibility using the inverse scaled snow depth was far too low. A final scaling was thus necessary to constrain the seasonal variability of these values so that they fell between the maximum value (*f* = 1) and an unknown but reasonable lower limit. For this, we use the following re-scaling calculation to get new values of the inverse scaled snow depth:

$$new value=\frac{\left( old value-old MIN \right)*\left( new Max-new Min \right)}{(old Max-old Min)} +new Min$$

where old value is the original inverse scaled snow depth, old min is 0, old max is 1, new max is 1, and new min is a parameter to estimate (${SD}_{thresh}$). The estimation of ${SD}_{thresh}$ was carried-out using a pattern-oriented modelling approach whereby a range of values were examined in order to see which produced results of seasonal body weight and reproductive success that matched observations. The result is a new inverse scaled snow depth, which follows the original daily and seasonal profile of snow depth, but now scaled to give predicted population data on seasonal body weights, reproductive success and survival that match observations. The functional response is then directly assigned the new value of this inverse scaled snow depth for each day of the year during the study period.

We did not include a snow-depth threshold at which animals could not access forage (i.e. cratering limit to food accessibility). Are model was not spatially explicit in resource availability nor animal movement/resource use, thus we assume a spatial and population mean representation of food provisioning whereby all animals can move to acquire some level of resources according to the snow depth effect on food quality and quantity.

## Sub-models

The following sub-models describe the processes carried-out at each time step. Model processes follow DEB theory and all fluxes are expressed in energy (J/time), mass in grams, and time in days (Figure 1). The DEB model equations are summarized in Table 2 and Table 3.

Table 2. Equations of the dynamic energy budget model for non-dependent individuals. Non-dependent individuals include juveniles and adults that assimilate energy entirely from the environment.

|  | **Equation** | **Unit** |
| --- | --- | --- |
| **State Variables** |  |  |
| Energy in reserve | $\frac{dE}{dt}=\dot{p}_{A}-\dot{p}_{C}$ | J |
| Structural length | $\frac{dL}{dt}= \frac{1}{3}\frac{\dot{p}_{G}}{\left[ E_{G} \right]L^{2}}$ | cm |
| Maturity | $\frac{dE_{H}}{dt}=\left\{ \begin{matrix} \dot{p}_{R}, \text{if} E_{H}< E_{H}^{p} \\ 0, \text{otherwise} \end{matrix} \right.$ | J |
| Reproduction buffer | $\frac{dE_{R}}{dt}=\left\{ \begin{matrix} 0, \text{if} E_{H}< E_{H}^{p} \\ \kappa_{R}\dot{p}_{R}, \text{otherwise} \end{matrix} \right.$ | J |
| **Energy Fluxes** |  |  |
| Assimilation | $\dot{p}_{A}$ $=f\left\{ \dot{p}_{Am} \right\}L^{2}$ | J |
| Reserve mobilization | $\dot{p}_{C}=E\cdot\frac{\dot{v}\left[ E_{G} \right]L^{2}+\dot{p}_{S}}{\kappa E+\left[ E_{G} \right]L^{3}}$ | J |
| Somatic maintenance | $\dot{p}_{S}=\left[ \dot{p}_{M} \right]L^{3}$ | J |
| Growth | $\dot{p}_{G}= \kappa\dot{p}_{C}-\dot{p}_{S}$ | J |
| Maturity maintenance | $\dot{p}_{J}=k_{J}E_{H}$ | J |
| Maturation and reproduction | $\dot{p}_{R}=(1-\kappa)\dot{p}_{C}-\dot{p}_{J}$ | J |
| Gestation | $\dot{G}_{cost}=\frac{\dot{p}_{F}}{\kappa_{R}}$ | J |
| Field metabolic rate (FMR) | $FMR= \dot{p}_{S}+\dot{p}_{J}+\dot{p}_{A}\left( 1-\kappa_{X} \right)+\dot{p}_{R}\left( 1-\kappa_{R} \right)+\dot{p}_{D}^{f}$ | J |

Table 3. Equations of the dynamic energy budget of the fetus.

|  | **Equation** | **Unit** |
| --- | --- | --- |
| **State Variables** |  |  |
| Energy in reserve | $\frac{dE^{f}}{dt}=\dot{p}_{E}^{f}-\dot{p}_{C}^{f}$ | J |
| Structural length | $\frac{dL^{f}}{dt}= \frac{\dot{v}^{f}}{3}$ | cm |
| Maturity | $\frac{dE_{H}}{dt}=\dot{p}_{R}^{f}$ | J |
| **Energy Fluxes** |  |  |
| Somatic maintenance | $\dot{p}_{S}^{f}=\left[ \dot{p}_{M} \right]{L^{f}}^{3}$ | J |
| Growth | $\dot{p}_{G}^{f}=\left[ E_{G} \right]\dot{v}^{f}{L^{f}}^{2}$ | J |
| Maturity maintenance | $\dot{p}_{J}^{f}=k_{J}E_{H}$ | J |
| Maturation | $\dot{p}_{R}^{f}=(1-\kappa)\dot{p}_{K}^{f}-\dot{p}_{J}^{f}$ | J |
| Kappa flux | $\dot{p}_{K}^{f}=\frac{\dot{p}_{S}^{f}+\dot{p}_{G}^{f}}{\kappa}$ | J |
| Reserve mobilization | $\dot{p}_{C}^{f}=E^{f}\cdot\frac{\dot{v}^{f}\left[ E_{G} \right]{L^{f}}^{2}+\dot{p}_{S}^{f}}{\kappa E^{f}+\left[ E_{G} \right]{L^{f}}^{3}}$ | J |
| Reproduction buffer to reserves | $\dot{p}_{E}^{f}=\dot{v}^{f}\left( 0.95 \left[ E \right] \right){L^{f}}^{2}+\dot{p}_{C}^{f}$ | J |
| Reproduction buffer to kappa | ${\dot{\dot{p}_{m}^{f}=p}}_{K}^{f}-\dot{p}_{C}^{f}$ | J |
| Total cost of gestation | $\dot{p}_{F}=\dot{p}_{m}^{f}+\dot{p}_{E}^{f}$ | J |
| Dissipated energy | $\dot{p}_{D}^{f}=\dot{p}_{K}^{f}- \kappa_{G} \dot{p}_{G}^{f}$ | J |

^a^ Only equations that differ from non-dependent are presented

### Temperature Correction

Empirical data of wild pregnant adult female muskoxen in high Arctic Greenland showed a relatively stable body temperatures at a mean of 38.2 ^o^C throughout winter and spring despite drastic changes in ambient temperature and snow cover. In contrast, body temperature in wild non-reproductive adult females fell throughout winter to a mean of 37.4 ^o^C and returned to ‘baseline’ levels in summer (Schmidt et al., 2020). We used the mean daily body temperature of normotherms and heterotherms and implemented individual variability around the mean in the model using the scatter-multiplier (see ‘Design Concepts’ for details). Heterothermic muskoxen were observed to have a greater range of daily $T_{B}$ than normotherms, thus the coefficients of variation were set at 0.15 and 0.10 respectively to arrive at observed levels of variability ($T_{B} x scatter-multiplier)$.

We assumed that the energy-saving of heterothermy arose from passive thermal effects of lowered $T_{B}$, via so called $Q_{10}$ or Arrhenius effects whereby the rates of enzymes are slowed at lower temperatures (Guppy and Withers, 1999). This is modelled via temperature correction factor ($TC$) applied to state parameters regulating metabolic rates (Kooijman, 2010). These included the volume-specific somatic maintenance rate ($\left[ \dot{p}_{M} \right]$), maturity maintenance rate constant ($\dot{k}_{J}$), and energy conductance ($\dot{v}$). $TC$ was modelled as a function of $T_{B}$, the Arrhenius temperature of the species ($T_{Ar}$), and a reference temperature ($T_{REF}$) following:

$$TC={exp}^{\frac{T_{Ar}}{T_{REF}}-\frac{T_{Ar}}{T_{B}}}$$

$T_{REF}$ is a reference temperature, commonly set to room temperature (293.15 K). The $T_{Ar}$ of muskoxen in unknown, thus we use the standard value in DEB theory that was used in the original calibration of the model (Desforges et al., 2019). We ran a sensitivity analysis to test the implications of this assumption (see ‘3.0 - Sensitivity Analysis’).

A fourth rate parameter in the model, namely the maximum specific assimilation rate ($\{\dot{p}_{Am}\}$) which determines the assimilation (i.e. ingestion) rate of the individual, could also be theoretically temperature corrected. Because it is unclear whether feeding follows Arrhenius effects similar to other metabolic processes, we tested both scenarios: 1) $\{\dot{p}_{Am}\}$ is independent of heterothermy (i.e., constant $\{\dot{p}_{Am}\}$); and 2) $\{\dot{p}_{Am}\}$ is body temperature dependent and thus feeding rates follow reductions in metabolic depression (i.e., temperature corrected $\{\dot{p}_{Am}\}$). The reasoning and implications of each strategy are discussed in the Results and Discussion of the main text of the manuscript. We ignore the influence of ambient temperature on metabolism as muskox are well adapted to freezing temperatures and most likely to be temperature stressed in the summer rather than winter (Munn et al., 2009).

### Assign feeding parameters

Daily snow depth values are read from external csv files and saved as lists within NetLogo. These raw data are then scaled and assigned to the functional response as described in ‘2.7 – Input data’.

### Reserve dynamics

This sub-model calculates the change in energy reserves ($E$) in a time step, which is simply the difference in assimilation ($\dot{p}_{A}$) and reserve mobilization ($\dot{p}_{C}$). Assimilation represents the energy entering the organisms from food after digestion and reserve mobilization describes the energy output from reserves used to fuel all metabolic processes.

$${\frac{dE}{dt}=\dot{p}}_{A}-\dot{p}_{C}$$

where $\dot{p}_{A}= f \left\{ \dot{p}_{Am} \right\}L^{2}$

$$\dot{p}_{C}= E \frac{\dot{v}\left[ E_{G} \right] L^{2}+\dot{p}_{S}}{\kappa E+\left[ E_{G} \right]L^{3}}$$

Somatic maintenance ($\dot{p}_{S}$) represents the costs associated with maintaining structural biomass in the organism and is proportional to structural volume and the volume-specific somatic maintenance rate

$$\dot{p}_{S}=\left[ \dot{p}_{M} \right] L^{3}$$

### Growth

Growth is defined as a change in structural length or volume$\left( V=L^{3} \right)$. The flux of energy allocated to growth ($\dot{p}_{G}$) is a fraction $\kappa$ of mobilized reserves ($\dot{p}_{C}$) and only what is left-over after somatic maintenance ($\dot{p}_{S}$) is paid

$$\dot{p}_{G}= \kappa\dot{p}_{C}-\dot{p}_{S}$$

The dynamics of structure is then a function of the growth flux and the volume-specific costs for growth

$$\frac{dL}{dt}= \frac{1}{3}\frac{\dot{p}_{G}}{\left[ E_{G} \right]L^{2}}$$

### Maturity & Reproduction Buffer

This sub-model describes the change in the reproduction buffer in adult females, and would include maturation in non-adult individuals if they were included in the model. A portion $1- \kappa$ of the mobilized reserve is used to pay maturity maintenance first ($\dot{p}_{J}$), and remaining energy ($\dot{p}_{R}$) goes to the reproduction buffer

$$\dot{p}_{R}=(1-\kappa)\dot{p}_{C}-\dot{p}_{J}$$

where $\dot{p}_{J}= k_{J}E_{H}$

### Reproduction (occurs in ‘initialize muskox population’)

Adult females are initiated as either pregnant or not using a drop-down chooser menu in the interface of the Netlogo model. If pregnant, the pregnancy status of the individual is set to true and the model recognizes the fetal development submodel (see below).

### Starvation

Starvation in DEB theory occurs when the fraction of mobilized reserves going to soma is insufficient to cover the costs of somatic maintenance (i.e. $\kappa\dot{p}_{C}<\dot{p}_{S}$). Specific rules are needed to dictate energy allocation if this should happen, and here these are set to prioritize somatic maintenance above all other metabolic processes. Since little is known about energy allocation during starvation in muskoxen, we implement the simplest approach possible, which follows observations that body fat reserves are used to fuel survival in winter (Adamczewski et al., 1995; White et al., 1997). For adults, we assume that individuals first divert exactly what is needed to cover somatic maintenance needs from the reproduction buffer ($E_{R}$). Next, if the reproduction buffer becomes unable to cover somatic maintenance, the missing energy is diverted directly from the reserves. This continues until the starvation status changes or the individual dies according to the rules established in the ‘Mortality’ sub-model.

### Update

The update sub-model is used to apply changes in the state and some auxiliary variables as well as perform some housekeeping on a daily time-step. First, reserves, structural length, reserve density, and scaled reserve density are updated (i.e. $E^{i+1}=E^{i}+\Delta E+ \dot{p}_{A\_E}^{+}$). Next the reproduction buffer is updated

$${E_{R}}^{i+1}={E_{R}}^{i}+ \dot{p}_{R}$$

For housekeeping purposes, rates of change in the primary state variables are reset to zero. Age is then updated so that at each daily time step a day is added to the age. Lastly, we calculate a suite of physical measurements, including physical length, dry weights of structure, reserves, reproduction buffer, and all combined, and physical wet weight. These are done using standard conversion values in DEB theory (Table 1).

### Fetal Development

While heterothermy was only observed in the small subset of non-pregnant animals in Schmidt et al. (2020), the presumed benefits of heterothermy extend to improved reproduction via higher energy reserves for reproduction (lactation) the following year. As per our systematic approach, we included pregnancy status as an additional hypothetical factor in the model design. As with non-pregnant females, we model normotherm and heterotherm temperature profiles as two groups of pregnant animals, all else being equal. We include two possible influences of heterothermy on reproduction: 1) embryo homeostasis is preserved and independent of maternal $T_{B}$, and 2) embryo development is a function of maternal $T_{B}$ and thus metabolic rates in the embryo energy budget are temperature corrected. Temperature correction was applied to the same rate parameters in the fetus as the mother.

The energy budget of the fetus is updated from the mother’s position; the sub-model is activated if the mother is pregnant. Fetal development (growth, etc.) commences only after an initial arrest period estimated to be 85 days ($t_{0}$). A schematic representation of the foetal DEB model is presented in Figure 1 and all parameters and energy fluxes detailed in Table 1 and Table 3. The fetus has the same DEB parameter values as the mother, with the exception of energy conductance ($\dot{v}^{f}$), which was estimated as a separate variable in DEBtool in order to fit model predictions with observations for fetal growth, lifetime growth, and ingestion rates. Somatic and maturity maintenance ($\dot{p}_{S}^{f}$ and $\dot{p}_{J}^{f}$) as well as reserve mobilization ($\dot{p}_{C}^{f}$) are calculated the same way as in other life-stages. Unlike the other life-stages, fetus energetics are demand processes driven by the needs for growth. Fetal structure grows linearly and proportional to energy conductance,

$$\frac{dL^{f}}{dt}= \frac{\dot{v}^{f}}{3}$$

and the growth flux becomes

$$\dot{p}_{G}^{f}=\left[ E_{G} \right]\dot{v}^{f}{L^{f}}^{2}$$

The sum of somatic maintenance and growth fluxes multiplied by $\kappa$ give the total energy flux allocated to soma ($\dot{p}_{K}^{f}$), or

$$\dot{p}_{K}^{f}= \frac{\dot{p}_{S}^{f}+\dot{p}_{G}^{f}}{\kappa}$$

What is left in the 1 – $\kappa$ branch after maturity maintenance is paid is allocated to maturity

$$\dot{p}_{R}^{f}=(1-\kappa)\dot{p}_{K}^{f}-\dot{p}_{J}^{f}$$

The dynamics of the flux coming from the mother to cover the full costs of the fetus depend on reserve mobilization dynamics as well as fetal costs of growth and maintenance. To capture a maternal effect where the healthy mothers develop healthy offspring and vice versa, we set the energy flux from the mother entering the reserves of the fetus ($\dot{p}_{E}^{f}$) proportional to the reserve density ($\left[ E \right]$) of the mother

$$\dot{p}_{E}^{f}=\dot{v}^{f}(0.95\cdot\left[ E \right]) {L^{f}}^{2}+\dot{p}_{C}^{f}$$

Though because newborns are typically more lean than their mothers (Adamczewski et al., 1995), we use a fraction of the maternal reserve density (95%) as the target for the foetus. Ultimately, the reserve of the fetus is the balance between input and output

$$\frac{{dE}^{f}}{dt}=\dot{p}_{E}^{f}-\dot{p}_{C}^{f}$$

The structure of the DEB model is similar to that in adults with reserve mobilization fueling metabolic processes following strong and weak homeostasis assumptions (Kooijman, 2010), however, in the fetus we add an additional flux ($\dot{p}_{m}^{f}$) that covers remaining metabolic needs if the reserve mobilization flux is insufficient

$$\dot{p}_{m}^{f}=\dot{p}_{K}^{f}-\dot{p}_{C}^{f}$$

thus providing the fetus with all the energy it requires to develop at maximum capacity. Ultimately, the total energy flux going to the fetus is given by

$$\dot{p}_{F}=\dot{p}_{m}^{f}+\dot{p}_{E}^{f}$$

The cost from the perspective of mother includes overhead costs of reproduction ($\kappa_{R}$), and is given by

$$\dot{G}_{cost}=\frac{\dot{p}_{F}}{\kappa_{R}}$$

The next step in this sub-model is similar to the ‘update’ sub-model in other life-stages, and is used to update all state variables with the estimated changes in that time step. Physical measurements, embryo age, and maturation are also updated here. The fetus is born when its maturity passes the birth maturity threshold. Lastly, the gestation costs are subtracted from the reproduction buffer of the mother; if the mother cannot cover the full costs with the energy available in her reproduction buffer, the missing energy is taken from her reserves.

### Mortality

Our model implementation includes two circumstances that trigger the death of an individual: starvation and fetal mortality.

Adult individuals increase their risk of starvation-related mortality as their body condition decreases, as measured by the scaled total reserve density ($e_{tot}$), following a logistic curve. $e_{tot}$ is the sum of reserves and reproduction buffer, scaled by structural volume to account for size, then scaled to the maximum reserve density (${[E]}_{max}=\frac{\left\{ \dot{p}_{Am} \right\}}{\dot{v}}$). The implementation of this body condition effect follows the assumption that animals in poor condition have an increased risk of mortality from other causes than old age and normal background mortality, for instance increased susceptibility to disease, competition for resources, and predation (Bender et al., 2007; De Roos et al., 2009; Loison et al., 1999). This risk is applied daily using a uniformly distributed value between 0 and 1 in comparison to the logistic mortality probability:

$$Survival prob= \frac{1}{(1+ {exp}^{{-k}_{s}\cdot(e_{tot}-starve\_coef})}$$

$$Mortality prob= 1-Survival prob$$

where $k_{s}$ and $starve\_coef$ are model parameters determining the shape of the survival curve. $k_{s}$ controls the steepness of the logistic curve, with large values resulting in steeper effects (Figure 2), while $starve\_coef$ controls the inflection point. Since no data was available to help determine the exact shape of the survival curve, we fixed $k_{s}$ at 30 in order to have good survival probability at higher body condition ($e_{tot}>0.8)$ and a gradual decline to the lower death threshold ($e_{tot}=0.20= e_{death})$. The death threshold value was chosen during model testing as it resulted in a final adult female body weight near observed weights of muskoxen that starved to death in Greenland (N. Schmidt, personal communication) and represents a second starvation-related mortality pathway.


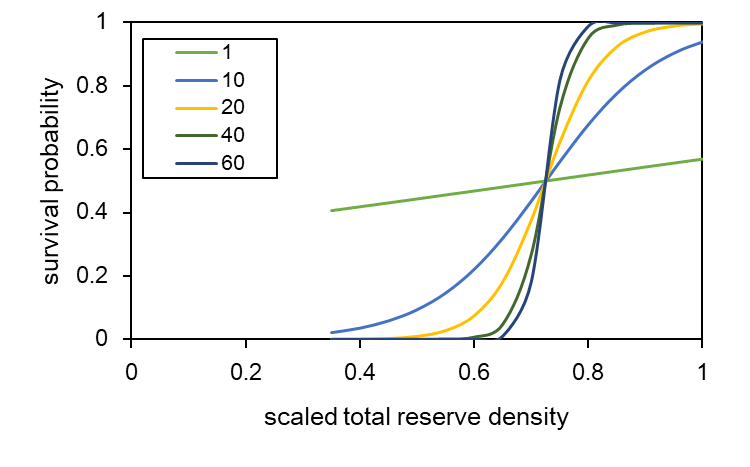


Figure 3. Survival probability curves as a function of total scaled reserve density. e_starv is kept constant at 0.75 to visualize effect of a change in k_starv from 1 to 60.

Fetal mortality is applied only during a brief 30 day window after the onset of active fetal development. This was identified as a sensitive window for muskox pregnancy (Rowell et al., 1993), and a likely time point to balance cost-benefits of reproduction. Here, the body condition of the mother dictates the probability of successful implantation following a logistic probability curve:

$$Implantation prob= \frac{1}{(1+ {exp}^{{-k}_{i}\cdot(e_{mom}-f\_surv\_coef})}$$

where $e_{mom}$ is the scaled total reserve density of the pregnant mother ($e_{tot}$), and $k_{i}$ and $f\_surv\_coef$ are model parameters determining the shape of the curve The parameter values of the logistic probability curve were determined by matching the shape of the implantation success curve against body weight and body fat percent in the muskox literature (Adamczewski et al., 1998; White, Rowell, & Hauer, 1997). These studies found a clear relationship between total body weight (and body condition) with the probability of successful pregnancy in October-November, which coincides closely with the beginning of active fetal development in our model. We apply this probability each day during a 30 day window which begins at the onset of active gestation (i.e. *t* = *t_0_*). The outcome of the equation is compared to a randomly generated number between 0 and 1, thus stochastically implementing fetal abortion. To find the relationship between body weight and scaled total reserve density, we ran our previous wild muskox model (Desforges et al. 2018) and plotted the predicted values at *t_0_* (Figure 5). These predicted $e_{tot}$ values were then used to parameterize the implantation probability equation, represented graphically in Figure 3.


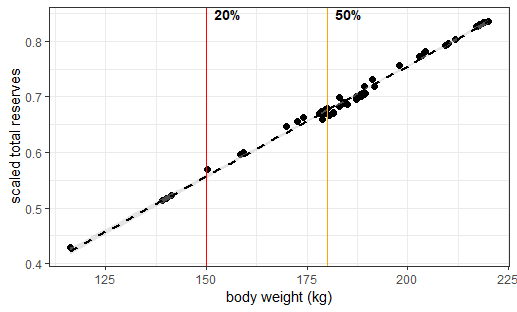


Figure 4. Modelled relationship between total body weight and scaled total reserve density of adult female muskoxen at time of implantation. Yellow and red vertical lines represent the body weights reported to cause 50% and 20% probability of pregnancy (Adamczewski et al., 1998).


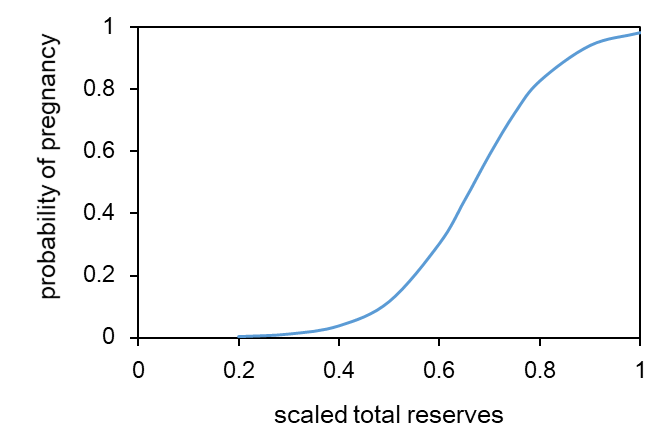


Figure 5. Graphical representation of the implantation probability equation. Probability of pregnancy is 1 – implantation prob.

# Sensitivity Analysis

We used the standard value of the Arrhenius temperature ($T_{A}$) in DEB theory since the value for muskoxen is unknown. To assess the implications of this assumption we conducted a simple sensitivity analysis by running the baseline model (i.e. compare heterotherms and normoatherms) for different values of $T_{A}$. The baseline value for $T_{A}$ used in DEB theory is 8000 K and this value has been used in the Add my Pet collection, which collates parameter values for thousands of species (https://www.bio.vu.nl/thb/deb/deblab/add_my_pet/). We test the impact of a higher and lower value, 7500 and 8500 K, on over-winter body weight, energy reserves, and survival. The results are shown in Figure 6. The difference between heterotherms and normotherms does not vary considerably between the tested values of $T_{A}$, while the magnitude of change from initial values and the variance for both groups increases with the value of $T_{A}$. There is no impact on survival. These results suggest that $T_{A}$ has important implications for the seasonal loss of body weight and energy reserves of all individuals, irrespective of metabolic strategy, but has little importance for the difference between individuals adopting metabolic strategies.

Figure 6. Sensitivity analysis for the Arrhenius temperature. Heterotherms and normotherms are shown in blue and orange, respectively.

# Model Scenarios

To assess the costs and benefits of heterothermy versus normothermy, we explore a range of physiological and environmental scenarios. Each scenario was repeated 25 times, and the recorded outputs included body weight, total energy reserves, and survival at the end of the model run as a percentage of initial values.


## Scenario 1

In scenario 1 we investigate the influence of a range of body temperatures on the difference in fitness output for heterotherms and normotherms. We use the same environmental input as the baseline model (i.e., same daily snow depth) but assess the influence of heterothermy at 0.1 ^o^C increments in daily body temperature from a plausible range of values (37.0-38.2 ^o^C) for ungulates.

## Scenario 2

In scenario 2 we investigate the influence of initial body condition on the over-winter outcome of heterotherm vs normotherm strategies. This was tested because autumn body condition varies considerably in wild muskoxen, with expected effects on reproductive success and survival. We assume the baseline body temperature profile of each group of animals, and initialize individuals at five percent increments of body reserves, starting at 100% (maximum reserves) and going down to 50% of maximum reserves.

## Scenario 3

In scenario 3 we investigate the influence of over-winter snow conditions on the outcome of heterotherm vs normotherm strategies. This was tested because snow conditions are known to vary considerably year to year and in the baseline we included only the average snow profile. We simulate changes in the daily snow depth simply by increasing or decreasing values of food availability (i.e. baseline functional response value, daily) by increments of ± 10%, again assuming the baseline body temperature profile of each group of animals.

## Temperature dependent feeding scenarios

To investigate the influence of feeding rate on the energetics and fitness of heterotherms and normotherms we included two possible scenarios. The first, and the primary scenario presented in the manuscript, is that the feeding parameter $\{\dot{p}_{Am}\}$ is not dependent on body temperature, thus feeding rates do not decrease with somatic maintenance (i.e., constant $\{\dot{p}_{Am}\}$). Here, $\{\dot{p}_{Am}\}$ is fixed throughout the model simulation at the initial value using a body temperature of 38.2 ^o^C ($\left\{ \dot{p}_{Am} \right\}=z \left[ \dot{p}_{M} \right] /\kappa$). The second scenario is that $\{\dot{p}_{Am}\}$ is dependent on body temperature, and thus feeding rates follow reductions in somatic maintenance (i.e., temperature corrected $\{\dot{p}_{Am}\}$). Here, $\{\dot{p}_{Am}\}$ is calculated at every time-step using the same equation as in the first scenario, but now $\left[ \dot{p}_{M} \right]$ changes as a function of body temperature via the temperature correction factor. The two contrasting scenarios were included because it is unclear which is occurring in wild ungulates. Baseline simulations and all scenarios were repeated for both feeding scenarios.

# Model Code

***Note: Model code below is formated for Netlogo v.6.02**

extensions [csv]

directed-link-breed [ parent-links parent ]

globals [

E_h^b T_REF L_0 p_A_m v_rate kap p_M

k_J k_M E_G kap_R kap_X kap_RL z

v_rate_f p_M_f k_J_f

d_V d_E mu_E mu_V w_E w_V diapause cv startpop

adult fetus year dayofyear mort.adult mort.fetus

snod_table snow snod_thresh snod_scaled_f outfile

hypo normo S1_factor

Sadd10 Sadd20 Sadd30 Sadd40 Ssub10 Ssub20 Ssub30 Ssub40

startPhysWeight startEE startER startEE_ER startEE_ER_d_scaled startFMR startpX startAdults startEmbryo startWHO

impPhysWeight impEE impER impEE_ER impEE_ER_d_scaled impFMR imppX impAdults impEmbryo

endPhysWeight endEE endER endEE_ER endEE_ER_d_scaled endFMR endpX endAdults endEmbryo endWHO endEmbryoWeight

]

turtles-own [

is-pregnant? EE dEE EE_max EEd EEd_scaled EEd_max scatter-multiplier

g E_R dE_R E_Rd EE_ER_d_scaled L dL EE_0 E_h dE_h

f p_X p_A p_C p_S p_G p_J p_R p_F p_E p_K p_MF GCost dGCost MetCost

age EmbryoAge lifestage PhysWeight DW_V DW_EE DW_R DW_TOT

p_A_m_sm calves-born mother_ID my_ID is-starved? FMR T TC

]

;; Setup procedure

to setup

reset-ticks

clear-ticks

clear-turtles

clear-patches

clear-drawing

clear-all-plots

clear-output

set E_h^b 7.1E05 set T_REF 293.15

set z 32.8 set L_0 0.0001 set E_G 7835 set kap 0.9779 set kap_R 0.95

set kap_X 0.3 set kap_RL 0.95

set d_V 0.3 set d_E 0.3 set mu_E 550000 set mu_V 500000 set w_E 23.9 set w_V 23.9

set cv 0.025 set diapause 85 set fetus 0 set adult 1 set startpop 25

load-data

Muskox-Initialization

set year 1 set dayofyear 270 ; October 1st set month 10

set mort.adult 0 set mort.fetus 0

set snod_thresh 0.70

;file-setup

reset-ticks

end

;; Temperature correction

to temp-correct

let TC_fix 4.72987 / 4.92945

; Scenarios for different pregnancy and hypometab status

ifelse scenario_temp = true ; true = run temperature gradient simulations, false = all other simulations

[

let scatter-multiplier-normo e ^ (random-normal 0 0.010)

set T (item ticks normo) * scatter-multiplier-normo

if status = "pregnant_H" or status = "nonpregnant_H" [ set T T * S1_factor ]

]

[

let scatter-multiplier-hypo e ^ (random-normal 0 0.015) ; more variation in hypo than normo

let scatter-multiplier-normo e ^ (random-normal 0 0.010)

if status = "pregnant_H" or status = "nonpregnant_H" [ set T (item ticks hypo) * scatter-multiplier-hypo ]

if status = "pregnant_NH" or status = "nonpregnant_NH" [ set T (item ticks normo) * scatter-multiplier-normo ]

]

set T T + 273.15

set TC e ^ ((T_A / T_REF) - (T_A / T))

set v_rate 0.07023 * TC_fix * TC

set p_M 91.76 * TC_fix * TC

set k_J 0.000000026 * TC_fix * TC

set k_M p_M / E_G

ifelse hypo_feed = TRUE [ set p_A_m z * p_M / kap ]

[ set p_A_m 14557.34 ]

ask turtles with [ lifestage = adult ] [ set p_A_m_sm p_A_m * scatter-multiplier ]

end

;; Initial Population Setup

to Muskox-Initialization

crt startpop

[

ifelse status = "pregnant_NH" or status = "pregnant_H" [ set is-pregnant? TRUE ]

[ set is-pregnant? FALSE ]

set lifestage adult

set scatter-multiplier e ^ (random-normal 0 cv)

;set p_A_m_sm p_A_m * scatter-multiplier

set EEd_max 43823.81 ;p_A_m / v_rate

set g E_G / (kap * EEd_max)

set L 31.5

set E_h 1.2E08

set age 2000

set EmbryoAge -1

set EE (condition * EEd_max) * (L ^ 3)

set EEd EE / (L ^ 3)

set EEd_scaled EEd / EEd_max

set EE_max (EEd_max) * (L ^ 3)

set E_R 0.0765 * EE + 127990205.3 ; derived from model testing

;set E_R 0.066 * EE - 9868609

set E_Rd E_R / (L ^ 3)

set EE_ER_d_scaled ((EE + E_R) / (L ^ 3)) / EEd_max

set p_F 0

set calves-born 0

set my_ID who set mother_ID -1

set is-starved? FALSE

set FMR -1

set MetCost 0

]

ask turtles with [ is-pregnant? = TRUE ] [ link-fetus ]

end

to load-data

file-close-all

set normo csv:from-file "normo.csv" ; temperature profile for normotherms

set normo reduce sentence normo

set hypo csv:from-file "hypo.csv" ; temperature profile for heterotherms

set hypo reduce sentence hypo

set snod_table csv:from-file "mean snow data.csv"

let snod-list reduce sentence snod_table

let snod_max max snod-list

let snod-list-scaled map [ m -> m / snod_max ] snod-list

let snod-list-scaled-2 map [ m -> 1 - m ] snod-list-scaled

set snow map [ m -> ((((m - 0) * (1 - snod_thresh)) / (1 - 0)) + snod_thresh) ] snod-list-scaled-2

set Sadd10 map [ m -> m * 0.9 ] snow set Sadd20 map [ m -> m * 0.8 ] snow

set Sadd30 map [ m -> m * 0.7 ] snow set Sadd40 map [ m -> m * 0.6 ] snow

set Ssub10 map [ m -> m * 1.1 ] snow set Ssub20 map [ m -> m * 1.2 ] snow

set Ssub30 map [ m -> m * 1.3 ] snow set Ssub40 map [ m -> m * 1.4 ] snow

end

;; GO procedure

to go

tick

set dayofyear dayofyear + 1

if dayofyear > 359 [ set dayofyear 0

set year year + 1 ]

ask turtles with [ lifestage = adult ] [ temp-correct

calc-Reserves

calc-Maturity-ReproBuffer

calc-Growth

calc-Starvation

calc-Mortality

Update ]

ask turtles with [ is-pregnant? = true ] [ calc-Fetal-Development ]

;ask turtles [ MakePlots ]

;ask turtles [ file-write-line ]

if dayofyear = 271 [ calc-start-ouput ]

if dayofyear = 359 [ calc-implant-ouput ] ; implant around 335, but export data after period of implant_check

if dayofyear = 119 [ calc-end-ouput ]

if not any? turtles [stop]

if ticks > 210 [ stop ] ; Model from Oct-May = 7 months = 210 days

end

;; Reserve dynamics

to calc-Reserves

if scenario = "baseline" [ set snod_scaled_f item ticks snow

set f snod_scaled_f ] ;* scatter-multiplier

if scenario = "add10" [ set snod_scaled_f item ticks Sadd10

set f snod_scaled_f ] ;* scatter-multiplier

if scenario = "add20" [ set snod_scaled_f item ticks Sadd20

set f snod_scaled_f ] ;* scatter-multiplier

if scenario = "add30" [ set snod_scaled_f item ticks Sadd30

set f snod_scaled_f ] ;* scatter-multiplier

if scenario = "add40" [ set snod_scaled_f item ticks Sadd40

set f snod_scaled_f ] ;* scatter-multiplier

if scenario = "sub10" [ set snod_scaled_f item ticks Ssub10

set f snod_scaled_f ] ;* scatter-multiplier

if scenario = "sub20" [ set snod_scaled_f item ticks Ssub10

set f snod_scaled_f ] ;* scatter-multiplier

if scenario = "sub30" [ set snod_scaled_f item ticks Ssub10

set f snod_scaled_f ] ;* scatter-multiplier

if scenario = "sub40" [ set snod_scaled_f item ticks Ssub10

set f snod_scaled_f ] ;* scatter-multiplier

set p_S p_M * (L ^ 3)

set p_A f * p_A_m_sm * (L ^ 2)

set p_C EE * (((v_rate * E_G * (L ^ 2)) + p_S) / ((kap * EE) + (E_G * (L ^ 3))))

set dEE p_A - p_C

set p_X p_A / kap_X

end

;; Maturity and reproduction

to calc-Maturity-ReproBuffer

set p_J k_J * E_h

set dE_h 0

set dE_R ((1 - kap) * p_C) - p_J

set p_R dE_R

end

;; Growth dynamics

to calc-Growth

set p_G (kap * p_C) - p_S

set dL (1 / 3) * p_G / (E_G * (L ^ 2))

if dL < 0 [ set dL 0 ]

end

;; Starvation

to calc-Starvation

ifelse ((p_C * kap) < p_S)

[

set is-starved? true

set p_G 0

set dL 0

let p_N1 p_S - kap * p_C

ifelse E_R > p_N1 [ set E_R E_R - p_N1 ]

[ let p_N2 p_N1 - E_R

set E_R 0

set dEE dEE - p_N2 ]

]

[

set is-starved? false

]

end

;; fetal development

to calc-Fetal-Development

ask in-parent-neighbors with [lifestage = fetus]

[ ifelse EmbryoAge > diapause ; start development after delayed implantation

[

let TC_fix 4.72987 / 4.92945

ifelse hypo-fetus = TRUE

[

; fetus is affected by hypometabolism

set T [T] of one-of out-parent-neighbors

set TC e ^ ((T_A / T_REF) - (T_A / T))

set v_rate_f 0.04648 * TC_fix * TC

set p_M_f 91.76 * TC_fix * TC

set k_J_f 0.000000026 * TC_fix * TC

]

[

; fetus not affected by hypometabolism

set T 38.2 + 273.15

set TC e ^ ((T_A / T_REF) - (T_A / T))

set v_rate_f 0.04648 * TC_fix * TC

set p_M_f 91.76 * TC_fix * TC

set k_J_f 0.000000026 * TC_fix * TC

]

set v_rate_f v_rate_f * (e ^ (random-normal 0 0.01))

set p_S p_M_f * (L ^ 3)

set p_J k_J_f * E_h

set p_G E_G * v_rate_f * (L ^ 2)

set p_K (p_S + p_G) / kap

set dE_h ((1 - kap) * p_K) - p_J

set dL v_rate_f / 3

if dL < 0 [ set dL 0 ]

let EEd_mom [EEd] of one-of out-parent-neighbors

set p_C EE * ((v_rate_f * E_G * (L ^ 2) + p_S) / ((kap * EE) + (E_G * (L ^ 3))))

set p_MF p_K - p_C

set p_E (v_rate_f * EEd_max * (L ^ 2)) + p_C ; EEd_mom

set dEE p_E - p_C

set dGCost (p_MF + p_E) / kap_R

let kap_G ((mu_V * d_V) / (w_V * E_G))

set MetCost (p_K - (kap_G * p_G))

; Update and reset variables

set L L + dL

set EE EE + dEE

set E_h E_h + dE_h

set EEd EE / (L ^ 3)

set EEd_scaled EEd / EEd_max

set E_R 0

set EE_max EEd_max * (L ^ 3)

set EE_ER_d_scaled ((EE + E_R) / (L ^ 3)) / EEd_max

;set EE_ER_d (EE + E_R) / (L ^ 3)

set GCost GCost + dGCost

set p_R dE_h

set EmbryoAge EmbryoAge + 1

set DW_V (L ^ 3) * d_V

set DW_EE EE * w_E / mu_E

set DW_R E_R * w_E / mu_E

set DW_TOT DW_V + DW_EE + DW_R

set PhysWeight DW_TOT / d_E

if E_h >= E_h^b [ ask one-of out-parent-neighbors [ set calves-born calves-born + 1 ]

die ] ; not keeping track of lactation

]

[ set EmbryoAge EmbryoAge + 1 ]

]

if any? in-parent-neighbors with [lifestage = fetus]

[

set p_F [dGCost] of one-of in-parent-neighbors with [lifestage = fetus]

set MetCost [ MetCost ] of one-of in-parent-neighbors with [lifestage = fetus]

ifelse E_R < p_F [ set EE EE - p_F ]

[ set E_R E_R - p_F ]

]

end

;; Mortality

to calc-Mortality

let k_s 30

let starve.coef 0.15

let surv.prob (1 / (1 + (e ^ ((k_s * -1) * (EE_ER_d_scaled - starve.coef)))))

let mort.prob (1 - surv.prob) ;/ 365

if random-float 1 < mort.prob

[

if any? in-parent-neighbors with [lifestage = fetus] [ set mort.fetus mort.fetus + 1

ask one-of in-parent-neighbors with [lifestage = fetus] [ die ] ]

set mort.adult mort.adult + 1

die

]

if EE_ER_d_scaled < 0.20

[

if any? in-parent-neighbors with [lifestage = fetus] [ set mort.fetus mort.fetus + 1

ask one-of in-parent-neighbors with [lifestage = fetus] [ die ] ]

set mort.adult mort.adult + 1

die

]

if any? in-parent-neighbors with [ lifestage = fetus ]

[ ask one-of in-parent-neighbors with [lifestage = fetus]

[

let e_MOM [ EE_ER_d_scaled ] of one-of out-parent-neighbors

let k_i 35

let implant_coef 0.625

let implant.prob (1 / (1 + (e ^ ((k_i * -1) * (e_MOM - implant_coef)))))

if (EmbryoAge > diapause) and (EmbryoAge < (diapause + 30))

[

if random-float 1 > (implant.prob) [ set dGCost 0

ask one-of out-parent-neighbors [ set is-pregnant? FALSE ]

set mort.fetus mort.fetus + 1

die

]

]

]

]

end

;; Fetus setup

to link-fetus

hatch 1

[

create-parent-to myself

set lifestage fetus

let hatch_ID who

let kid self

set mother_ID my_ID

set my_ID who

set EmbryoAge 20 set age 0 ; start 20 days later than usual conception

set L L_0 set dL 0

let EEd_mom [EEd] of one-of out-parent-neighbors

set EE_0 (L_0 ^ 3) * EEd_mom

set EE EE_0

set E_R 0

set EEd EE / (L ^ 3)

set EEd_scaled 0

set EE_max EEd_max * (L ^ 3)

set EE_ER_d_scaled ((EE + E_R) / (L ^ 3)) / EEd_max

set dEE 0 set E_h 0 set dE_h 0 set dE_R 0

set E_Rd E_R / (L ^ 3)

set is-pregnant? FALSE

set is-starved? FALSE

set calves-born 0

set FMR -1

set MetCost 0

set T 0

set TC 0

set scatter-multiplier e ^ (random-normal 0 cv) ; cv set to 5%

set p_A_m_sm p_A_m * scatter-multiplier

set p_A 0 set p_C 0 set p_E 0 set p_G 0 set p_S 0 set p_K 0

set dGCost 0 set GCost 0 set p_J 0 set dE_h 0 set dL 0

set DW_V (L ^ 3) * d_V

set DW_EE EE * w_E / mu_E

set DW_R 0

set DW_TOT DW_V + DW_EE + DW_R

set PhysWeight DW_TOT / d_E

]

end

;; Update

to Update

;--------- Increase state variables ---------------------------------

set EE EE + dEE

set L L + dL

set EEd EE / (L ^ 3)

set EEd_scaled EEd / EEd_max

set E_R E_R + p_R

set E_Rd E_R / (L ^ 3)

set EE_max EEd_max * (L ^ 3)

set EE_ER_d_scaled ((EE + E_R) / (L ^ 3)) / EEd_max

set FMR p_S + p_J + (p_R * (1 - kap_R)) + (p_A * (1 - kap_X)) + MetCost

set age age + 1

;--------- reset derivatives - just in case --------------------------

set dEE 0 set dL 0 set dE_h 0 set dE_R 0

;-------- calculate physical measurements-----------------------------

set DW_V (L ^ 3) * d_V

set DW_EE EE * w_E / mu_E

set DW_R E_R * w_E / mu_E

set DW_TOT DW_V + DW_EE + DW_R

set PhysWeight DW_TOT / d_E

end

;; Save population data

to calc-start-ouput

set startWHO [ WHO ] of turtles with [ lifestage = adult ]

set startPhysWeight [ PhysWeight ] of turtles with [ lifestage = adult ]

set startEE [ EE ] of turtles with [ lifestage = adult ]

set startER [ E_R ] of turtles with [ lifestage = adult ]

set startEE_ER [ EE + E_R ] of turtles with [ lifestage = adult ]

set startEE_ER_d_scaled [ EE_ER_d_scaled ] of turtles with [ lifestage = adult ]

set startFMR [ FMR ] of turtles with [ lifestage = adult ]

set startpX [ p_X ] of turtles with [ lifestage = adult ]

set startAdults ((count turtles with [ lifestage = adult ]) / startpop) * 100

set startEmbryo ((count turtles with [ lifestage = fetus ]) / startpop) * 100

end

to calc-implant-ouput

set impPhysWeight [ PhysWeight ] of turtles with [ lifestage = adult ]

set impEE [ EE ] of turtles with [ lifestage = adult ]

set impER [ E_R ] of turtles with [ lifestage = adult ]

set impEE_ER [ EE + E_R ] of turtles with [ lifestage = adult ]

set impEE_ER_d_scaled [ EE_ER_d_scaled ] of turtles with [ lifestage = adult ]

set impFMR [ FMR ] of turtles with [ lifestage = adult ]

set imppX [ p_X ] of turtles with [ lifestage = adult ]

set impAdults ((count turtles with [ lifestage = adult ]) / startpop) * 100

set impEmbryo ((count turtles with [ lifestage = fetus ]) / startpop) * 100

end

to calc-end-ouput

set endWHO [ WHO ] of turtles with [ lifestage = adult ]

set endPhysWeight [ PhysWeight ] of turtles with [ lifestage = adult ]

set endEE [ EE ] of turtles with [ lifestage = adult ]

set endER [ E_R ] of turtles with [ lifestage = adult ]

set endEE_ER [ E_R + EE ] of turtles with [ lifestage = adult ]

set endEE_ER_d_scaled [ EE_ER_d_scaled ] of turtles with [ lifestage = adult ]

set endFMR [ FMR ] of turtles with [ lifestage = adult ]

set endpX [ p_X ] of turtles with [ lifestage = adult ]

set endAdults ((count turtles with [ lifestage = adult ]) / startpop) * 100

set endEmbryo ((count turtles with [ lifestage = fetus ]) / startpop) * 100

set endEmbryoWeight [ PhysWeight ] of turtles with [ lifestage = fetus ]

end

# References

Adamczewski, J., Fargey, P.J., Laarveld, B., Gunn, A., Flood, P.F., 1998. The influence of fatness on the likelihood of early-winter pregnancy in muskoxen. Theriogenology 50, 605–614.

Adamczewski, J., Flood, P., Gunn, A., 1995. Body composition of muskoxen ( Ovibos moschatus ) and its estimation from condition index and mass measurements. Can. J. Zool. 73, 2021–2023.

Adamczewski, J., Flood, P.F., Gunn, A., 1997. Seasonal patterns in body composition and reproduction of female muskoxen (Ovibos moschatus). J. Zool. 241, 245–269. doi:10.1111/j.1469-7998.1997.tb01956.x

Arnold, W., Ruf, T., Kuntz, R., 2006. Seasonal adjustment of energy budget in a large wild mammal, the Przewalski horse (Equus ferus przewalskii) II. Energy expenditure. J. Exp. Biol. 209, 4566–4573. doi:10.1242/jeb.02536

Bender, L., Lomas, L., Browning, J., 2007. Condition, Survival, and Cause-Specific Mortality of Adult Female Mule Deer in North-Central New Mexico. J. Wildl. Manage. 71, 1118–1124. doi:10.2193/2006-226

Brinkmann, L., Gerken, M., Riek, A., 2012. Adaptation strategies to seasonal changes in environmental conditions of a domesticated horse breed, the Shetland pony (Equus ferus caballus). J. Exp. Biol. 215, 1061–1068. doi:10.1242/jeb.064832

De Roos, A.M., Galic, N., Heesterbeek, H., 2009. How resource competition shapes individual life history for nonplastic growth: Ungulates in seasonal food environments. Ecology 90, 945–960. doi:10.1890/07-1153.1

Desforges, J.-P., Marques, G.M., Beumer, L.T., Chimienti, M., Blake, J., Rowell, J.E., Adamczewski, J., Schmidt, N.M., van Beest, F.M., 2019. Quantification of the full lifecycle bioenergetics of a large mammal in the high Arctic. Ecol. Modell. 401, 27–39. doi:10.1016/j.ecolmodel.2019.03.013

Falk, J.M., Schmidt, N.M., Christensen, T.R., Ström, L., 2015. Large herbivore grazing affects the vegetation structure and greenhouse gas balance in a high arctic mire. Environ. Res. Lett. 10, 45001. doi:10.1088/1748-9326/10/4/045001

Gaillard, J., Yoccoz, N.G., Loison, A., Toigo, C., 2000. Temporal variation in fitness components and population dynamics of large herbivors. Annu. Rev. Ecol. Syst. 31, 367–393.

Geiser, F., 2004. Metabolic Rate and Body Temperature Reduction During Hibernation and Daily Torpor. Annu. Rev. Physiol. 66, 239–274. doi:10.1146/annurev.physiol.66.032102.115105

Grimm, V., Berger, U., Bastiansen, F., Eliassen, S., Ginot, V., Giske, J., Goss-Custard, J., Grand, T., Heinz, S.K., Huse, G., Huth, A., Jepsen, J.U., Jørgensen, C., Mooij, W.M., Müller, B., Pe’er, G., Piou, C., Railsback, S.F., Robbins, A.M., Robbins, M.M., Rossmanith, E., Rüger, N., Strand, E., Souissi, S., Stillman, R. a., Vabø, R., Visser, U., DeAngelis, D.L., 2006. A standard protocol for describing individual-based and agent-based models. Ecol. Modell. 198, 115–126. doi:10.1016/j.ecolmodel.2006.04.023

Guppy, M., Withers, P., 1999. Metabolic depression in animals: Physiological perspectives and biochemical generalizations. Biol. Rev. 74, 1–40. doi:10.1111/j.1469-185X.1999.tb00180.x

Helle, T., Kojola, I., 2008. Demographics in an alpine reindeer herd: Effects of density and winter weather. Ecography (Cop.). 31, 221–230. doi:10.1111/j.0906-7590.2008.4912.x

Kooijman, S., 2010. Dynamic Energy Budget theory for metabolic organisation, 3rd editio. ed. Cambridge University Press. doi:10.1098/rstb.2010.0167

Liston, G.E., Elder, K., 2006a. A Meteorological Distribution System for High-Resolution Terrestrial Modeling (MicroMet). J. Hydrometeorol. 7, 217–234. doi:10.1175/JHM486.1

Liston, G.E., Elder, K., 2006b. A Distributed Snow-Evolution Modeling System (SnowModel). J. Hydrometeorol. 7, 1259–1276. doi:10.1175/JHM548.1

Loison, A., Langvatn, R., Solberg, E.J., 1999. Body mass and winter mortality in red deer calves: Disentangling sex and climate effects. Ecography (Cop.). 22, 20–30. doi:10.1111/j.1600-0587.1999.tb00451.x

Marn, N., Jusup, M., Legović, T., Kooijman, S.A.L.M., Klanjšček, T., 2017. Environmental effects on growth, reproduction, and life-history traits of loggerhead turtles. Ecol. Modell. 360, 163–178. doi:10.1016/j.ecolmodel.2017.07.001

Martin, B.T., Zimmer, E.I., Grimm, V., Jager, T., 2012. Dynamic Energy Budget theory meets individual-based modelling: A generic and accessible implementation. Methods Ecol. Evol. 3, 445–449. doi:10.1111/j.2041-210X.2011.00168.x

Mosbacher, J.B., Michelsen, A., Stelvig, M., Hendrichsen, D.K., Schmidt, N.M., 2016. Show me your rump hair and i will tell you what you ate - The dietary history of muskoxen (ovibos moschatus) revealed by sequential stable isotope analysis of guard hairs. PLoS One 11, 1–13. doi:10.1371/journal.pone.0152874

Mosbacher, J.B., Michelsen, A., Stelvig, M., Hjermstad-Sollerud, H., Schmidt, N.M., 2018. Muskoxen Modify Plant Abundance, Phenology, and Nitrogen Dynamics in a High Arctic Fen. Ecosystems. doi:10.1007/s10021-018-0323-4

Munn, A.J., Barboza, P.S., Dehn, J., 2009. Sensible Heat Loss from Muskoxen ( *Ovibos moschatus* ) Feeding in Winter: Small Calves Are Not at a Thermal Disadvantage Compared with Adult Cows. Physiol. Biochem. Zool. 82, 455–467. doi:10.1086/605400

Pedersen, S.H., Liston, G.E., Tamstorf, M.P., Abermann, J., Lund, M., Schmidt, N.M., 2018. Quantifying snow controls on vegetation greenness. Ecosphere 9, e02309. doi:10.1002/ecs2.2309

Riek, A., Brinkmann, L., Gauly, M., Perica, J., Ruf, T., Arnold, W., Hambly, C., Speakman, J.R., Gerken, M., 2017. Seasonal changes in energy expenditure, body temperature and activity patterns in llamas (Lama glama). Sci. Rep. 7, 7600. doi:10.1038/s41598-017-07946-7

Rowell, J.E., Pierson, R.A., Flood, P.F., 1993. Endocrine changes and luteal morphology during pregnancy in muskoxen (Ovibus moschatus). J. Reprod. Fertil. 99, 7–13. doi:10.2307/3504280

Ruf, T., Geiser, F., 2015. Daily torpor and hibernation in birds and mammals. Biol. Rev. 90, 891–926. doi:10.1111/brv.12137

Schmidt, N.M., Pedersen, S.H., Mosbacher, J.B., Hansen, L.H., 2015. Long-term patterns of muskox (Ovibos moschatus) demographics in high arctic Greenland. Polar Biol. 38, 1667–1675. doi:10.1007/s00300-015-1733-9

Signer, C., Ruf, T., Arnold, W., 2011. Hypometabolism and basking: The strategies of Alpine ibex to endure harsh over-wintering conditions. Funct. Ecol. 25, 537–547. doi:10.1111/j.1365-2435.2010.01806.x

Turbill, C., Ruf, T., Mang, T., Arnold, W., 2011. Regulation of heart rate and rumen temperature in red deer: effects of season and food intake. J. Exp. Biol. 214, 963–970. doi:10.1242/jeb.052282

White, R., Rowell, J., Hauer, W., 1997. The role of nutrition, body condition and lactation on calving success in muskoxen. J. Zool. 243, 13–20.
